# The Properties and Role of *O*-Acyl-ω-hydroxy Fatty Acids and Type I-St and Type II Diesters in the Tear Film Lipid Layer Revealed by a Combined Chemistry and Biophysics Approach

**DOI:** 10.1021/acs.joc.0c02882

**Published:** 2021-03-17

**Authors:** Tuomo Viitaja, Jan-Erik Raitanen, Jukka Moilanen, Riku O. Paananen, Filip S. Ekholm

**Affiliations:** † Department of Chemistry, University of Helsinki, P.O. Box 55, FI-00014 Helsinki, Finland; ‡ Ophthalmology, University of Helsinki and Helsinki University Hospital, Haartmaninkatu 8, FI-00290 Helsinki, Finland

## Abstract

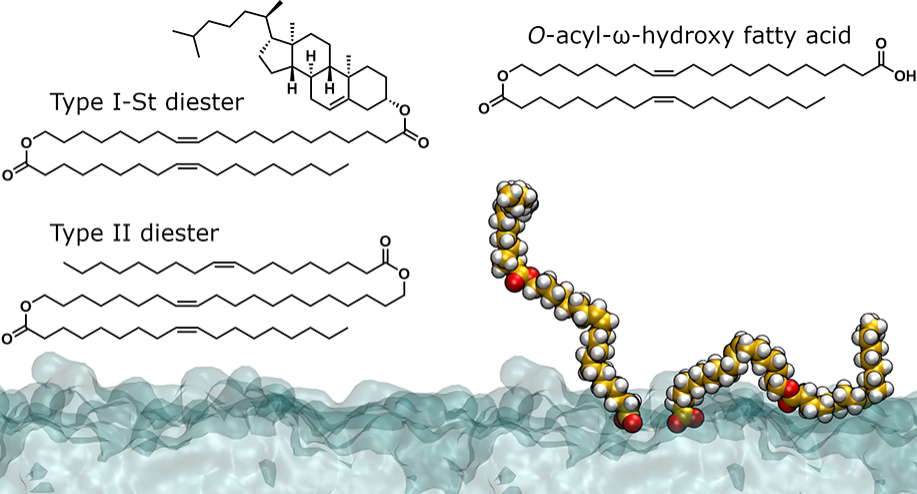

The tear film lipid layer (TFLL) that covers the ocular surface contains several unique lipid classes, including *O*-acyl-ω-hydroxy fatty acids, type I-St diesters, and type II diesters. While the TFLL represents a unique biological barrier that plays a central role in stabilizing the entire tear film, little is known about the properties and roles of individual lipid species. This is because their isolation from tear samples in sufficient quantities is a tedious task. To provide access to these species in their pure form, and to shed light on their properties, we here report a general strategy for the synthesis and structural characterization of these lipid classes. In addition, we study the organization and behavior of the lipids at the air–tear interface. Through these studies, new insights on the relationship between structural features, such as number of double bonds and the chain length, and film properties, such as spreading and evaporation resistance, were uncovered.

## 1 Introduction

**Figure 1:**
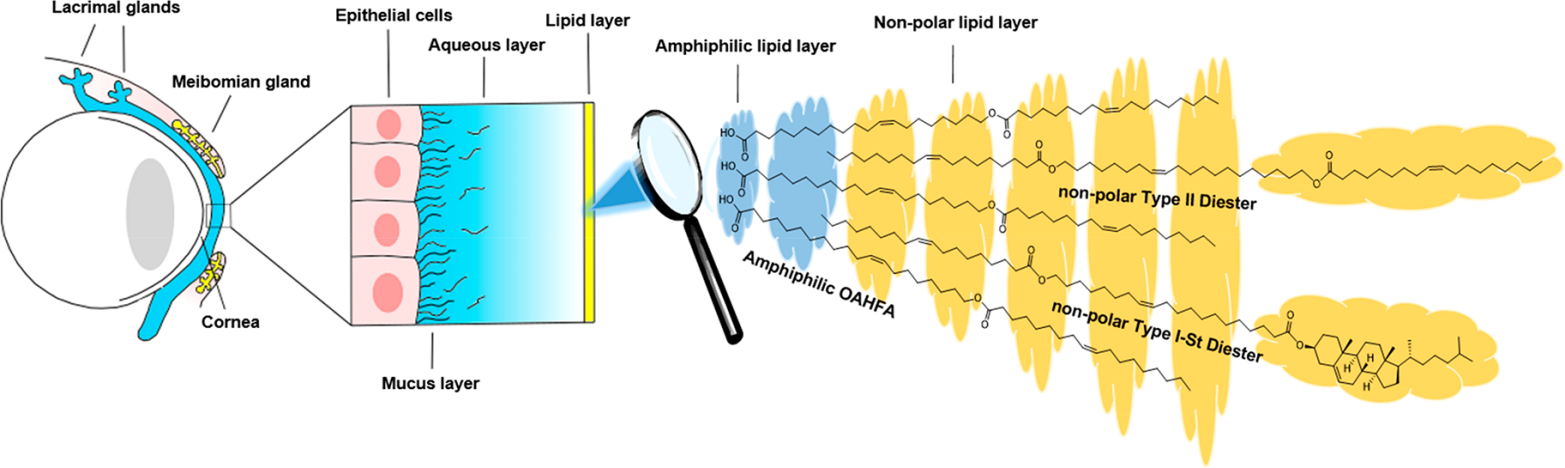
A schematic presentation of the ocular surface and the structure of the tear film with a further emphasis on the organization of the TFLL and TFLL lipid classes, which are the focus of this study.

The tear film lipid layer (TFLL) resides on top of the tear film, where it covers the ocular surface and protects and lubricates the surface of the eye (Figure 1). It is a specialized lipid membrane secreted by the Meibomian glands, forming a barrier that, according to the traditional view, prevents the evaporation of aqueous tear fluid. However, the evaporation-resistant function is still poorly understood.^1^ In recent years, there has been a growing interest in understanding the structure and function of the TFLL. This is due to an increased awareness of the relationship between its intrinsic properties and the maintenance of ocular surface health. Most notably, a compromised TFLL function is closely related to ocular surface diseases such as dry eye disease (DED), which affects more than 500 million people on a global scale^2^ and constitutes a significant public health concern and societal economic burden.^3,4^ A common characteristic of DED is the drying of the ocular surface due to the excess evaporation of tear fluid,^5^ a condition that is presumably linked to structural changes in the TFLL composition.^6−9^ Addressing the properties of individual TFLL lipids is thus important to understand the molecular-level basis of DED. The TFLL contains several unique classes of lipids that are characterized by ultralong unsaturated hydrocarbon chains, including *O*-acyl-ω-hydroxy fatty acids (OAHFAs) and type I-St (cholesteryl) and type II (wax) diesters (Figure 1).^10^

Although each of these special lipid classes constitutes a relatively small fraction (3–4 mol % each) of the total lipids in Meibomian gland secretions, recent results have suggested that they may be central to TFLL function.^11−14^ OAHFAs have been proposed to be potential indicators of DED progression, and a consistent decrease in the number of OAHFA species has been correlated to DED severity in patient-derived meibum samples.^15^ While a positive correlation between the Schirmer I test and the tear film breakup time for the total OAHFA levels has been noted,^14^ uncertainties still remain since statistically significant decreases in total tear OAHFA levels has not been confirmed in DED patients.^15^ Nevertheless, reduced levels of type I-St and type II diesters have been reported in patient-derived tear samples compared to those in healthy controls.^16^ Moreover, animal studies have shown that the knockout of the Cyp4f39-gene involved in the synthesis of OAHFAs and their related diesters in the Meibomian glands leads to the development of a severe dry eye phenotype in mice.^17^ Therefore, there are indications that OAHFAs and their diester derivatives may be central to proper TFLL function. However, the physical properties and molecular behavior of these lipid classes have not previously been studied in detail and thus open questions regarding their role in TFLL function and organization remain.

Biophysical studies on OAHFAs and diesters have been hindered by their limited availability. The isolation of these lipid species, devoid of biological contaminations, from the natural source has proved to be challenging due to variations in the lipid composition between species and individuals, the minute quantities of tear samples that are extractable from humans, and the large number of lipid species present in the TFLL. As a result, simplified structural analogues have been used in previous biophysical studies.^18,19^ Our recent study addressing the the biophysical properties of OAHFA and type II diester analogues revealed that OAHFAs are capable of forming evaporation-resistant monolayers at the air–tear interface.^19^ While these results were promising, the structural analogues studied deviated from the naturally occurring TFLL lipids, which contain an additional *cis*-double bond in one of the hydrocarbon chains.^20^

We therefore decided to build on our previous work and investigate the effects of an additional double bond on the biophysical properties of the lipids. This required a complete revision of the synthetic strategy and the development of synthetic routes for the synthesis of an OAHFA and its related type I-St and type II diesters. To the best of our knowledge, there are only a handful of reports^18−22^ on the synthesis of type II diesters and OAHFAs in the literature and no reports on the synthesis of type I-St diesters. Furthermore, the majority of the previous studies that address the synthesis and characterization of OAHFAs suffer from ambiguities concerning the synthetic protocols applied and, even more surprisingly, the supplied characterization data is often lacking. In this work, we have therefore invested a great deal of effort into both these aspects and provide a block approach applicable to the synthesis of the majority of the unique TFLL lipids and their structural analogues. In addition to the synthesis, the most detailed NMR spectroscopic characterization data reported to date are supplied. These methods were used to synthesize a model OAHFA, a type I-St diester, and a type II diester. With the pure TFLL model lipids in hand, we studied their biophysical properties and evaporation resistance with Langmuir monolayer techniques. Our results indicate that the disordering effect of the additional double bond in OAHFAs likely balances the ultralong chain lengths in naturally occurring OAHFAs to promote spreading on the tear film surface. In addition, type I-St diesters were found to exhibit a liquid-crystalline structure at the aqueous interface, suggesting their involvement in the multilamellar organization of the TFLL.

## 2 Results and Discussion

### 2.1 Total Synthesis of Tear Film Lipid Probes

**Figure 2:**
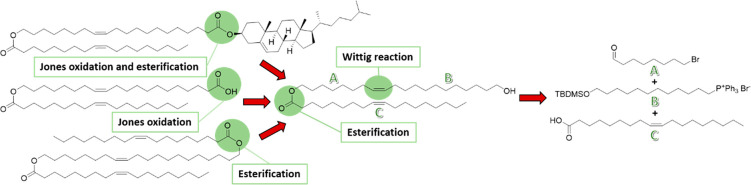
Retrosynthetic analysis on which the designed synthetic route to produce 20:1-OAHFA, 20:1-DiE, and 20:1-St-DiE was based. The three starting fragments (A, B, and C), the core intermediate, and the products are shown with the key organic transformations highlighted.

The successful construction of complex organic molecules, such as the three TFLL lipid model compounds targeted in this study, requires a sound and tailored synthetic strategy. We therefore performed a detailed retrosynthetic analysis, which revealed that the OAHFA and its type I-St and type II diesters can all be constructed from one common core intermediate (see Figure 2). The core intermediate can be prepared from three separate fragments, which are denoted A, B, and C.

It is important to note that this general strategy is applicable to the preparation of a large number of naturally occurring TFLL-lipids and their unnatural analogues by varying the chain lengths and structural features of fragments A, B, and C. In this work, we focused solely on the preparation of a short 20:1-OAHFA and its type I-St cholesteryl and type II oleoyl diesters. A shorter version of the naturally occurring OAHFAs was targeted to examine the effect of the double bond, since we have recently studied the properties of the corresponding 20:0-OAHFA and found them to be interesting.^19^ Considering that the naturally occurring lipids almost exclusively have a (*Z*)-configuration around the double bond,^20^ we envisioned that the highly (*Z*)-selective Wittig olefination reaction would be a suitable protocol to generate the required stereochemistry in a coupling reaction between fragments A and B. For the construction of the core intermediate, we further chose to use oleic acid as the representative fragment C since it is the most abundant acyl chain found in these classes of TFLL-lipids.^11,12^ From the core intermediate onward, we envisioned that mild oxidation and esterification reactions would be required to avoid unwanted transesterification side reactions and the oxidation of the double bonds. With this retrosynthetic analysis as a base, we set out to synthesize the OAHFA analogue and the related diesters.

**Scheme 1:**
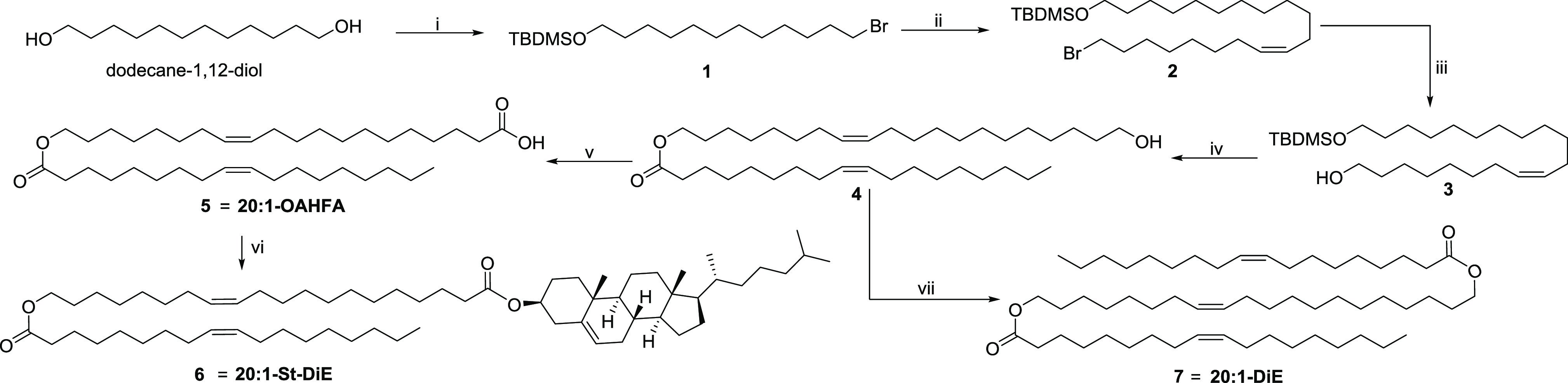
Overview of the Reaction Routes and Isolated Yields (i) (1) 48% aq HBr, cyclohexane, reflux, 18 h, 77%; (2) imidazole, TBDMSCl, CH_2_Cl_2_, rt, o/n, 94%. (ii) (1) PPh_3_, neat, 120 °C, 17 h, quant.; (2) NaHMDS, dry THF, HMPA, −78 °C, 1 h, then 8-bromo-1-octanal, −78 °C, rt, 24 h, 34%. (iii) (1) KOAc, DMSO, 50 °C, 27 h, 66%; (2) NaOMe, THF/MeOH (1:2), 22 h, rt, 78%. (iv) (1) Oleic acid, DMAP, EDC·HCl, CH_2_Cl_2_, rt, o/n, 93%; (2) TBAF, THF, 1 h, rt, 92%. (v) Jones reagent, acetone/EtOAc (1:1), 0 °C, 45 min, 89%. (vi) Cholesterol, DMAP, EDC·HCl, CH_2_Cl_2_, rt, o/n, 67%. (vii) Oleic acid, DMAP, EDC·HCl, CH_2_Cl_2_, rt, o/n, 70%.

Our synthetic route began with the preparation of fragment B, and the routes to all three target lipids are displayed in Scheme 1. In the first step, commercially available 1,12-dodecanediol was successfully monobrominated according to a protocol reported earlier by Greaves et al.^23^ The monobrominated 12-bromo-1-dodecanol could be isolated in a 77% yield in a reaction using a large excess of HBr in cyclohexane. The yield is well within the 65–92% range commonly reported in the literature.^24−26^ To access compound **1**, the temporary protection of the hydroxyl group as a TBDMS-ether was required. The TBDMS-ether was chosen because of its stability toward basic conditions and the mild and selective installation and deprotection protocols available in the literature.^27^ Surprisingly, the installation of the TBDMS-ether featuring a small excess of TBDMSCl (1.3 equiv) led to a 3:4 mixture of the desired product and an unknown side product. By the careful characterization of the compound mixture by NMR spectroscopy, the second product was determined to be 12-chloro-1-*tert*-butyldimethylsilyloxydodecane based on the chemical shift of C-12 (45 vs 34.1 ppm in **1**). To the best of our knowledge, this type of halide–halide displacement reaction has not previously been reported to take place under the employed reaction conditions, although the reaction protocols applied by other groups suggest that similar problems likely have been previously encountered.^28^ To circumvent this side reaction, the reaction solvent was changed from dry DMF to dry CH_2_Cl_2_ and the amount of TBDMSCl was reduced from 1.3 to 0.8 equiv, while 2 equiv of imidazole was used. These minor adjustments to the reaction protocol, which resembles the protocol employed by Cateni et.al.,^29^ led to the exclusive formation of **1** in an excellent 94% yield. To access fragment B, **1** was reacted with 1 equiv of PPh_3_ at 120 °C under neat reaction conditions. While similar reactions are commonly performed in refluxing acetonitrile^30^ or toluene, an additional solvent was not required.^29^ To confirm that fragment B had formed, i.e., the phosphonium bromide salt, ^31^P NMR spectra were recorded, and the disappearance of the PPh_3_ signal at −5.4 ppm and the appearance of the signal at 24.4 ppm were monitored. When the conversion was complete, the crude product was used as such in the Wittig olefination reaction. The second fragment required for the Wittig reaction, i.e., fragment A, was prepared by the selective oxidation of commercial 8-bromo-1-octanol with PCC to afford 8-bromo-octanal in an 84% yield. The successful formation of the aldehyde was confirmed by ^1^H NMR spectroscopy, and all signals were assigned. After workup and drying, fragments A and B were immediately coupled in a (*Z*)-selective Wittig reaction according to a modified procedure to give **2** in a 34% yield.^31^ In more detail, the required phosphonium ylide was formed *in situ* with NaHMDS at −78 °C before the coupling with fragment A was performed. The low yield observed is due to the use of crude starting materials and the sluggish reaction in which a large number of side products were formed. As a result, the chromatographic separation of **2** was challenging and required multiple tedious column purifications before a pure product could be isolated. The direct conversion of **2** to **3** with H_2_O in DMF at elevated temperatures was attempted but proved unsuccessful, and a two-step protocol was devised based on the earlier work of Lee et al.^32^ The bromide was first displaced with an acetyl group in a 66% isolated yield using a large excess of KOAc in DMSO. The purified compound was then deacetylated under Zemplén^33^ conditions to afford **3** in a 77% yield. We initially attempted to perform this two-step process according to the one-pot protocol reported by Lee et.al.;^32^ however, satisfactory results with our substrate could not be obtained. To access the core intermediate **4**, esterification with oleic acid was required. We chose to avoid the use of Fischer esterification with sulfuric acid, which may isomerize the double bonds,^34^ and sought inspiration from the previously published work of Balas et al.^22^

We settled on the use of a Steglich-type esterification protocol with the minor modification of replacing DCC with EDC·HCl. Under these conditions, the esterification proceeded smoothly in a 93% yield. The temporary silyl protective group was deprotected with 3 equiv of TBAF in an excellent yield. From this core intermediate, the 20:1-OAHFA (**5**) could be prepared in an 89% yield by a mild oxidation with the Jones reagent according to our previously established reaction protocol.^19^ The Jones oxidation is an especially useful protocol for these lipids because alkene functionalities are unaffected by the reaction conditions.^35^ The use of EtOAc as a cosolvent is rare in the literature, but we have found that it has a beneficial effect on the reaction outcome and is somewhat superior to the other cosolvents typically applied (water or THF). With the 20:1-OAHFA prepared, we completed the synthesis of the related cholesteryl type I-St diester **6** (20:1-St-DiE) by a Steglich-type esterification with cholesterol in a 67% yield following the previously described protocol. The oleoyl type II diester **7** (20:1-DiE) was prepared from **4** and oleic acid in a similar fashion in a 70% yield.

In summary, the three TFLL model lipids **5**–**7** were obtained in 10 or 11 synthetic steps in overall yields ranging between 5–7.5%. All synthesized products, with the exception of fragments A and B, were purified by conventional techniques and characterized in detail by NMR spectroscopy (1D and 2D techniques), which were further coupled with quantum-mechanical spectral simulations with the PERCH (PEak reseaRCH) software and HRMS. Owing to the lack of characterization data reported for these compounds in the literature, a further segment on the NMR spectroscopic characterization is provided below.

### 2.2 Complete NMR Spectroscopic Characterization of the Tear Film Lipid Probes

**Figure 3:**
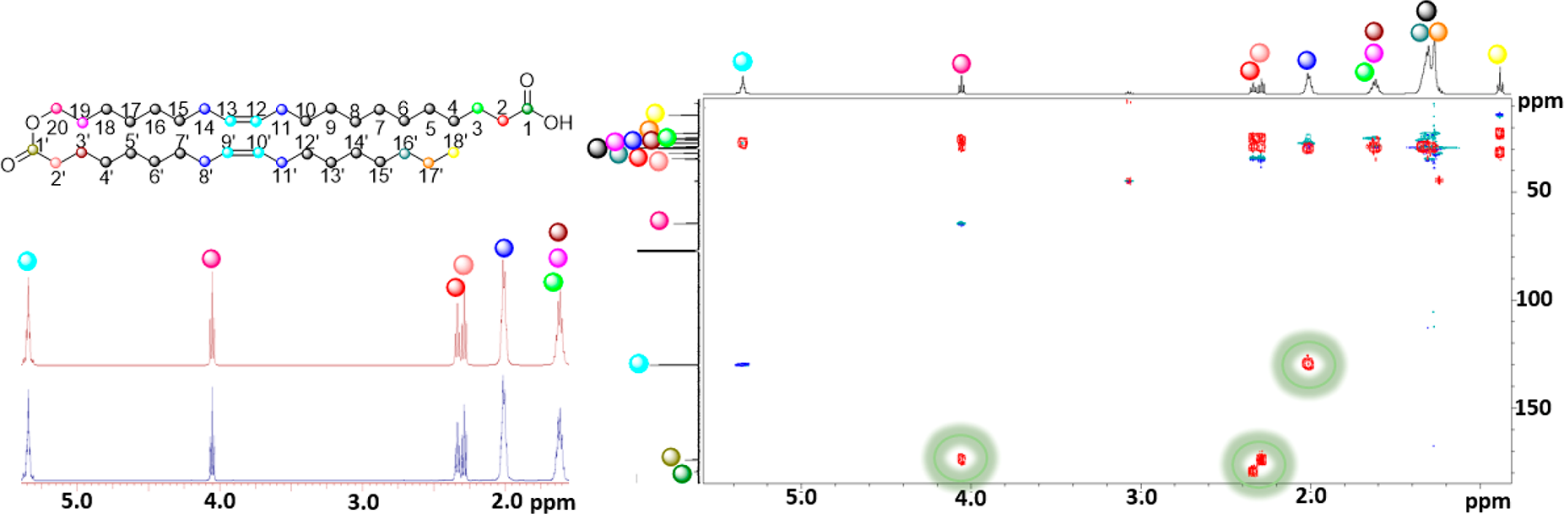
(Left) The numbering of the atoms in 20:1-OAHFA is displayed in the molecular structure along with the colors used for the visualization of the different signals in the spectra (top) and the 5.3–1.5 ppm region of the ^1^H NMR spectrum that highlights the accuracy of the spectral simulations with the PERCH software (the measured spectrum is in blue and the simulated spectrum is in red) (bottom). (Right) Overlapping Ed-HSQC (CH and CH_3_ are in blue and CH_2_ in green) and HMBC (red) spectra, with the most important HMBC correlation highlighted with green circles.

In the literature, many of the previous synthetic routes to OAHFAs are accompanied by a lack of structural characterization data to varying degrees. In some cases, only TLC *R*_f_ values, FT-IR, and mass data are provided.^18,21^ In other publications, NMR analysis was performed but, for example, the ^13^C NMR data were not assigned, and the determination of the stereochemistry in key intermediates and end products leaves room for improvement.^20^ In this work, great effort was invested in the structural characterization of the end products by NMR spectroscopic techniques, and a detailed review of the characterization flow is warranted. While all molecules were characterized to the greatest extent possible, the 20:1-OAHFA (**5**) will be used as the sole example here. The numbering of the molecule and an excerpt of the key methods used in the NMR spectroscopic characterization are summarized in Figure 3.

The NMR spectroscopic characterization began with the identification of the carbonyl carbons at 179.6 (C-1) and 174.2 ppm (C-1′). The observed HMBC correlations between C-1′–H-20, C-1′–H-2′, and C-1–H-2 highlighted in Figure 3 were used to ascertain that these two signals were appropriately assigned and the acyl chain was located at the correct position. With H-2 (t at 2.34 ppm), H-20 (t at 4.05 ppm), and H-2′ (t at 2.29 ppm) identified and their corresponding carbons assigned through the use of Ed-HSQC, H-3 and C-3 (t at 1.63 ppm and s at 24.9 ppm, respectively), C-18 (s at 26.1 ppm), H-19 and C-19 (tt at 1.61 ppm and s at 28.8 ppm, respectively) and H-3′ and C-3′ (t at 1.61 ppm and s at 25.2 ppm, respectively) could be assigned by a combination of COSY, Ed-HSQC, and HMBC.

The second starting point in the characterization was the characteristic chemical shifts of the alkene functionalities (5.35–5.34 ppm in ^1^H and 130.1–129.9 ppm in ^13^C) and the signals in close proximity to them (2.06–1.96 ppm in ^1^H and 27.4–27.3 ppm in ^13^C). These signals could not be assigned by conventional techniques due to their severe overlap, and moreover the coupling patterns were found to be complex and higher-order in their nature. Yet, coupling constants for H-12–H-13 and H-9′–H-10′ were required to ensure a (*Z*)-configuration in the double bonds. Therefore, we used the quantum-mechanical spectral simulation software PERCH,^36^ which can be used to extract chemical shifts and coupling constants in the presence of overlapping signals and higher order effects.^37−40^ With the aid of spectral simulations, we were able to extract the chemical shifts of the alkenes and, more importantly, all the coupling constants in the complex dtt patterns could be defined. The coupling constants for the *vicinal* protons in the alkenes were determined to be *J*_9′,10′_ = 11.1 Hz and *J*_12,13_ = 11.6 Hz, which are in agreement with the literature values given for the (*Z*)-configuration,^41^ thus confirming the correct stereochemistry of the alkene moieties. The third starting point used in the spectral characterization was at the H-18′ and C-18′ signals, which appear at characteristic chemical shifts (t at 0.88 ppm and s at 14.3 ppm, respectively). By applying the conventional techniques listed above, the C-16′ and H-17′ and C-17′ signals could be assigned. All in all, we were able to solve all the well-resolved signals in the ^1^H and ^13^C NMR spectra and a number of the overlapping signals with complex coupling patterns, as displayed in Figure 3 and further reported in the Experimental Section in more detail. The regions where multiple signals overlapped, i.e., 1.37–1.23 and 29.9–29.3 ppm, could not be solved in detail, but all signals that occurred in these regions could nevertheless be identified. The accurate reference chemical shifts for the OAHFA and its diesters reported herein provide a valuable foundation for future NMR spectroscopic characterization studies of lipids isolated from Meibomian gland secretion or the synthesized analogues of these unique TFLL lipids. In addition, the chemical shifts reported provide a base for the development of NMR-based lipidomic profiling tools.^42−45^ With the synthesis and accurate characterization studies completed, we turned our attention to the investigation of the biophysical properties of these lipids to shed light on their role in the TFLL.

### 2.3 Biophysical Properties of OAHFAs and Related Diesters

The TFLL can be divided into an amphiphilic sublayer, which resides at the interface between the aqueous tear film and the TFLL, and an overlying nonpolar sublayer (see Figure 1). Due to their hydrophobic nature, diesters are expected to exist in the nonpolar sublayer, while amphiphilic OAHFAs are expected to reside in the amphiphilic sublayer. However, as outlined in the introduction, the detailed biophysical properties of tear film OAHFAs and diesters have been poorly characterized, and several open questions regarding their role in TFLL organization and function remain. Understanding the biophysical properties and molecular behavior of the species represent an important and hitherto missing link in refining our view of the factors that influence the structure and function of the TFLL.

The synthesized OAHFA and diesters were designed to function as excellent molecular probes of TFLL lipids. We were therefore expecting to uncover new insights on the role and behavior of these lipid classes in the TFLL while simultaneously being able to correlate the results to previous reports with simplified structural analogues.^18,19^ To this end, we studied their organization at the aqueous interface using a Langmuir trough system equipped with surface-potential and surface-pressure sensors, a Brewster angle microscope (BAM), and a custom Langmuir–Schaefer-type^46^ setup to measure the evaporation resistance. Briefly, the lipids were spread on the surface of a phosphate-buffered saline (PBS) subphase maintained at 35 °C to mimic the conditions of the ocular surface. The lipid film was compressed and expanded using Delrin barriers to simulate the compression occurring during blinking, and the properties of the lipid film were measured at different compression states. Reversible compression–expansion cycles were observed for all the synthesized lipids (Figure S26).

### 2.3.1 20:1-OAHFA

**Figure 4:**
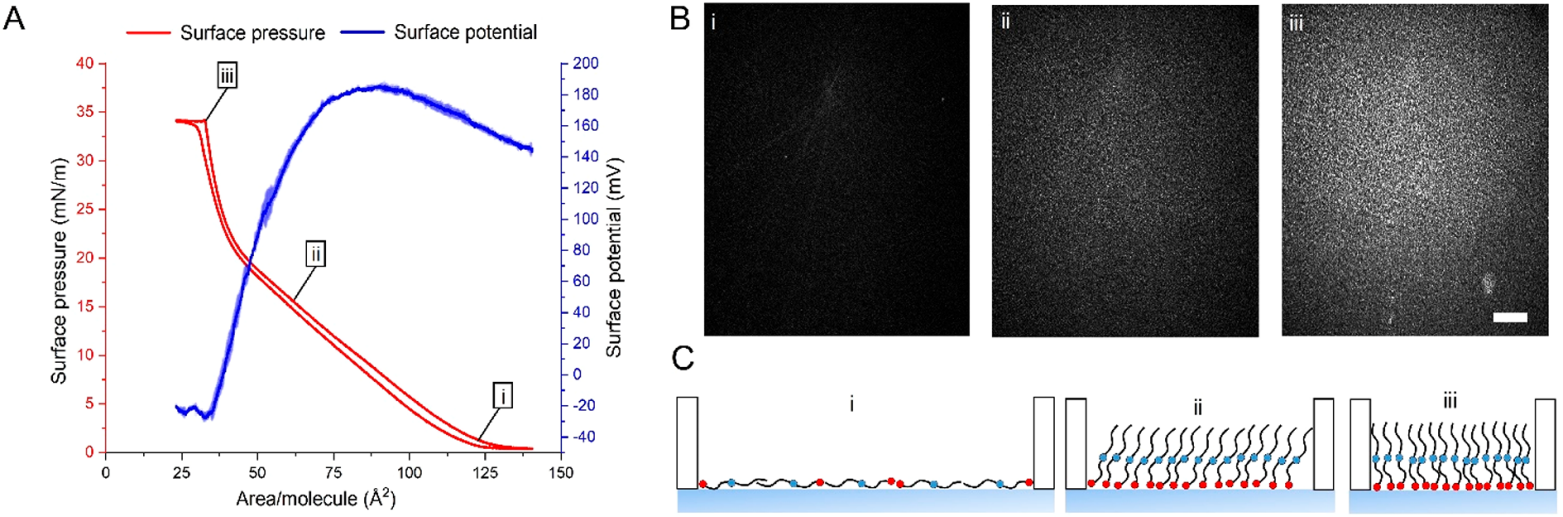
Organization of 20:1-OAHFA at the aqueous interface. (A) A representative surface-pressure compression–expansion cycle and the mean surface potential (±SD, *n* = 3) of the 20:1-OAHFA monolayers at 35 °C. (B) The corresponding BAM images. In the BAM images, the pure PBS surface appears black, and the local image intensity increases according to the thickness of the lipid film on the surface (also shown in Figure 5 and 6). The scale bar depicts 500 μm. (C) Schematic representation of the molecular organization of the 20:1-OAHFA films during compression, with red and blue circles representing the carboxylic and ester groups, respectively, and lines representing the hydrocarbon chains.

20:1-OAHFA (**5**) spread readily and formed a monolayer on the PBS subphase. Upon compression, the surface pressure started to increase at a mean molecular area of around 130 Å^2^/molecule and the film appeared homogeneous in BAM images (Figure 4A and Bi), thereby implying a disordered monolayer organization. The isotherm lift-off area was similar to our previously reported results with the analogous 20:0-OAHFA.^19^ The results suggest that 20:1-OAHFA adopts a disordered conformation at low surface pressures in which it lies flat on the aqueous interface with the carboxylic acid and ester groups interacting with the aqueous sublayer (Figure 4Ci).

When compressed to smaller areas per molecule, the molecules gradually adopted an upright orientation (Figure 4Cii) as previously described.^18,19^ This change in orientation can be observed as a decrease in the surface potential (Figure 4A). Despite this change in orientation, the 20:1-OAHFA monolayer remained in a disordered phase in contrast to saturated OAHFAs of a corresponding length, which underwent a transition to a condensed monolayer phase at a surface pressure of 2 mN/m.^19^ This finding is supported by the homogeneous appearance of the films in the BAM images, although the reflected intensity increased with the increasing surface pressure, indicating a gradual increase in the thickness of the film (Figure 4Bii and Biii). When the film was compressed to 40 Å^2^/molecule, a second-order phase transition was observed in the 20:1-OAHFA surface-pressure isotherm that manifested as a steep increase in the slope (Figure 4A). This phase transition potentially reflects a change in the conformation of the 20:1-OAHFA from a tilted alignment to a horizontal alignment (Figure 4Ciii), which is in line with previous reports on fatty acid monolayers.^47^ However, the 20:1-OAHFA monolayer remained disordered even in this phase since the formation of solid domains was not observed in the BAM images (Figure 4Biii). In addition, the film collapse occurred at 30 Å^2^/molecule, which is significantly larger than the mean molecular area of most condensed monolayers (20 Å^2^/molecule).^19,47^

The presence of an additional double bond in 20:1-OAHFA therefore prevented the formation of a condensed monolayer phase at high surface pressures similar to those in natural tears (27–31 mN/m).^48^ The absence of a solid monolayer phase was accompanied by a lack of evaporation resistance (see the Supporting Information), which is in line with our previous study.^19^ The disordered structure presented here describes the behavior of the shortest OAHFAs detected in Meibomian gland secretions and tears.^11,15^ Despite the lack of evaporation resistance, these OAHFAs may contribute to stabilizing and spreading the nonpolar TFLL lipids through their surfactant properties.

In addition, the organization of the most abundant OAHFAs in the TFLL with even longer hydroxy fatty acid (HFA) chains (30:1 to 34:1)^11,15,20^ can be estimated by comparing the results to our earlier work.^19^ The overall organization of the 20:1-OAHFA closely resembled that of the shorter 12:0-OAHFA;^19^ therefore, the disordering effect caused by the presence of a second *cis*-double bond is similar in magnitude to an eight-carbon decrease of the HFA chain length. Therefore, the ultralong OAHFAs with unsaturated HFA chains in the TFLL would be expected to organize similarly to the 20:0-OAHFA, and we consider it to be likely that they are capable of forming a solid monolayer with evaporation-resistant properties;^19^ however, this needs to be confirmed by future work.

### 2.3.2 Type II 20:1-Diester

Type II diesters belong to a hydrophobic lipid class and are therefore expected to reside in the nonpolar sublayer of the TFLL. Interestingly, they have been found to appear in reduced amounts in DED patients.^16^ Their role in the TFLL function is not clear, but they have been suggested to stabilize the TFLL by connecting the amphiphilic and nonpolar sublayers.^17^ Therefore, understanding their surface behavior and properties is important.

**Figure 5:**
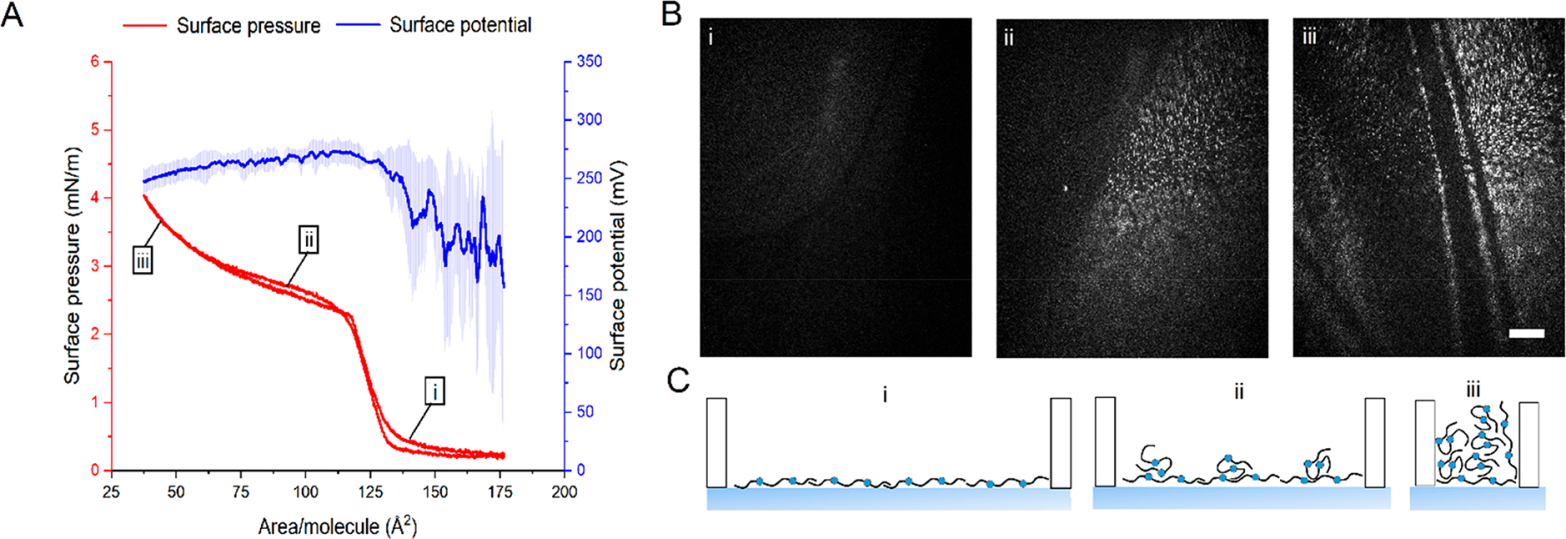
Organization of 20:1-DiE at the aqueous interface. (A) A representative surface-pressure compression–expansion cycle and the mean surface potential (±SD, *n* = 3) of the 20:1-DiE monolayer at 35 °C. (B) Corresponding BAM images. The scale bar depicts 500 μm. (C) Schematic representation of the molecular organization of the 20:1-DiE films during compression.

The oleoyl type II diester (**7**, 20:1-DiE) was found to spread on the aqueous interface in a similar fashion to the 20:1-OAHFA (Figure 5A). The surface-pressure isotherm lift-off was observed at 130 Å^2^/molecule, and BAM images indicated the formation of a homogeneous monolayer (Figure 5Bi). On a general level, the surface-pressure and surface-potential isotherms of 20:1-DiE closely resembled previously studied type II diesters featuring a saturated interconnecting diol moiety.^19^ Therefore, the likely configuration of 20:1-DiE molecules in the monolayer involves both ester groups facing the water interface and the oleoyl chains oriented toward the air in a disordered fashion (Figure 5Ci). Such a disordered low-density monolayer did not result in a detectable evaporation resistance (see the Supporting Information). When compressed, plateaus in the surface pressure and surface potential were observed that were accompanied by the appearance of high-intensity aggregates in the BAM images (Figure 5Bii and Biii), indicating that the film collapsed at a surface pressure of 2 mN/m. Although the collapse surface pressure was low, a stable monolayer was formed, indicating that the presence of a *cis*-double bond in the diol moiety of 20:1-DiE facilitated spreading on the aqueous surface. The presence of an additional double bond thus changes the behavior of the DiE to a certain extent, since the corresponding saturated 20:0-DiE was incapable of forming a stable monolayer even at 43 °C as reported in our previous work.^19^

The collapse pressure of 20:1-DiE was close to that of a saturated 15:0-DiE,^19^ indicating that the addition of a *cis*-double bond in the diol moiety had a similar effect on the diester spreading properties as a five-carbon decrease in the diol chain length. Based on these observations and previous results, the spreading properties of the most-abundant naturally occurring type II diesters in tear fluid with 30–34 carbons in the interconnecting diol chain^12,49^ can be estimated. In our series of synthetic probes, the collapse surface pressure of the diesters was found to decrease by 0.7 mN/m for each one-carbon increase in the diol chain length.^19^ Therefore, an extension of the 20:1-DiE diol chain with 10–14 carbon atoms (representing naturally occurring type II diesters) would not result in a type II diester with monolayer-forming capabilities due to the predicted zero equilibrium spreading pressure.

### 2.3.3 Type I 20:1-St Diester

**Figure 6:**
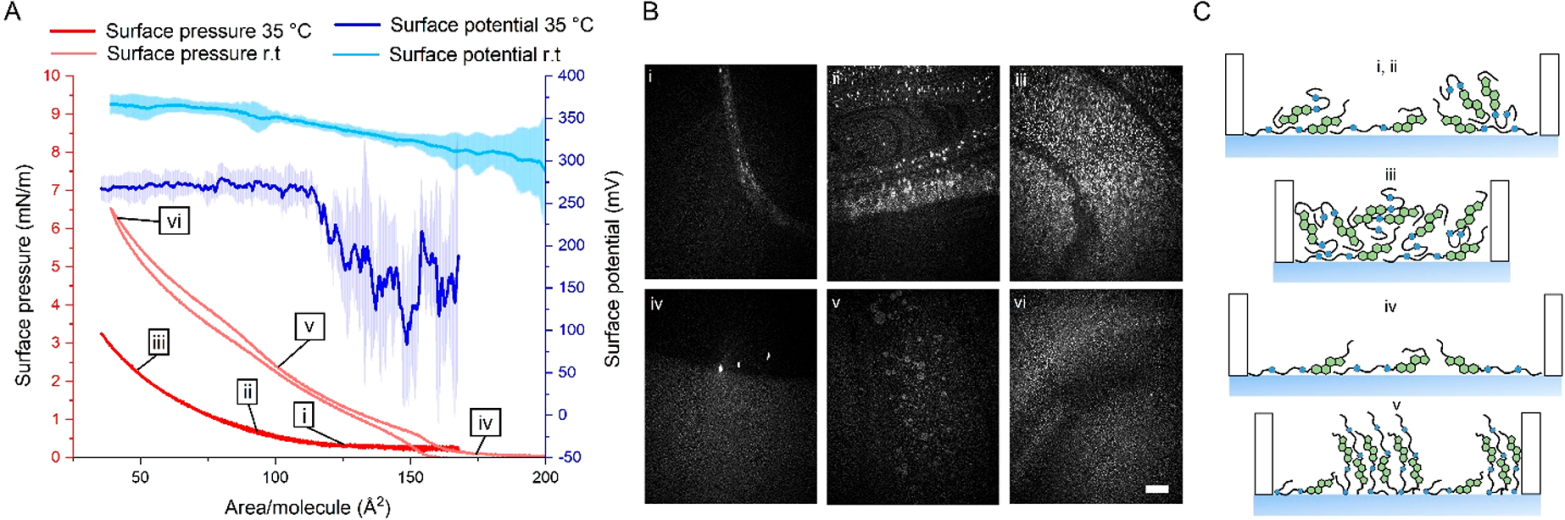
(A) Surface pressure and surface potential (mean ± SD, *n* = 3) of 20:1-St-DiE with (B) the corresponding BAM images at 35 °C and room temperature. The scale bar depicts 500 μm. (C) Schematic representation of the molecular organization of the 20:1-St-DiE films during compression at 35 °C and room temperature.

While type I-St diesters have been known to exist in the TFLL for decades,^50^ little is known about their properties due to the lack of molecular probes from this lipid class. Here we studied 20:1-St-DiE, which is a shorter analogue of the TFLL type I-St diesters. In contrast to 20:1-DiE, 20:1-St-DiE only partially spread on the PBS surface at 35 °C, as indicated by the lack of surface pressure (Figure 6A) and the high-intensity aggregates dispersed throughout the monolayer in the BAM images (Figure 6Bi–Biii). The reduced surface activity compared to that of 20:1-DiE can be explained by the bulkier nature of the nonpolar cholesteryl moiety, which leads to stronger intramolecular van der Waals interactions between the diesters and thus favors aggregation. The 20:1-St-DiE films lacked evaporation-resistant properties at 35 °C (see the Supporting Information), which can be attributed to the incomplete spreading and disordered structure of the film (Figure 6Ci–Ciii).

We therefore decided to further investigate whether the surface organization of 20:1-St-DiE is affected by the temperature. Interestingly, 20:1-St-DiE spread to form a monolayer at room temperature but not at 40 °C (see Figure S25). This was evidenced by a shift of the surface-pressure and surface-potential isotherms to larger areas per molecule (Figure 6A and B). In addition, a homogeneous monolayer was observed in the BAM images at room temperature (Figure 6Biv). A surface potential of approximately 300 mV was observed at the surface-pressure lift-off similar to 20:1-DiE, thus suggesting that the conformation of 20:1-St-DiE at the aqueous interface may be similar with both ester groups facing the water interface (Figure 6Civ). Upon the compression of the monolayer, the surface pressure started to increase at approximately 150 Å^2^/molecule, reflecting the larger size of 20:1-St-DiE compared to that of 20:1-DiE. The formation of circular higher-intensity domains with a uniform intensity appeared during compression (Figure 6Bv). This was interesting because phase transitions were not apparent from the recorded isotherm data. Moreover, the appearance of these domains resembled the formation of multilamellar structures reported to occur in mixed films containing polar lipids and cholesteryl nervonate, where layers of the cholesteryl ester form above the polar lipid monolayer.^51^ Therefore, we propose a putative organization for 20:1-St-DiE in which the diesters adopt an extended conformation similar to that of cholesteryl ester multilayers but with the second ester groups oriented toward the water (Figure 6Cv). Unfortunately, the details of their organization could not be determined based on these results alone. When the films were compressed to even smaller areas (<80 Å^2^/molecule), the observed domains were compressed into bright droplets, which is suggestive of a collapse of the lipid film (Figure 6Bvi). The absence of a plateau in the surface-pressure isotherm poses a challenge for a more detailed analysis of the structural features of the film. Further work is currently under planning to address the structural features of these type I-St-DiEs in more detail. At this stage, it is sufficient to state that 20:1-St-DiE appeared to form a liquid-crystalline phase similar to the multilayers formed by cholesteryl esters and the results suggest that the type I-St diester may be involved in the formation of multilamellar structures in the TFLL.

## 3 Conclusion

Herein we described the chemical synthesis, detailed NMR-spectroscopic characterization, and investigation of the biophysical properties of a model tear fluid OAHFA, a type I-St diester, and a type II diester species. We have devised a functioning synthetic strategy that permits the preparation of these lipid classes from one core intermediate. The most important benefits of the chosen synthetic strategy are that the block approach can be applied to the synthesis of a variety of TFLL lipids and structural analogues thereof by varying the chemical structures of fragments A, B, and C (see Figure 2). When this information is further coupled with the most-detailed NMR spectroscopic characterization data reported for these compounds to date, a critical tool for the future synthesis work and NMR spectroscopic lipidomic profiling of the tear-film emerges.

The biophysical characterization described provides a general description of the surface behavior of these unique lipid species, offering insights on their role in the TFLL under states of health and disease. Monounsaturated OAHFA analogues were recently demonstrated to form an evaporation-resistant solid monolayer at the aqueous interface, and the evaporation resistance was found to improve with an increase in the HFA chain length.^19^ However, as increasing the chain length also markedly decreases the spreading rate of the surfactants,^52,53^ a compromise between chain length and spreadability is likely essential to achieve effective evaporation resistance. Herein, we have showed that an additional double bond in the HFA chain was accompanied by a more disordered molecular organization of the OAHFAs, which led to the loss of the evaporation resistance. Therefore, it seems likely that the ultralong chain lengths observed in naturally occurring OAHFAs require the presence of an additional double bond to achieve an appropriate balance between spreadability and evaporation resistance.

Diesters have been suggested to reside between the polar OAHFAs and the nonpolar wax and cholesteryl esters due to their intermediate polarity.^17^ However, based on our results both diester classes display very limited surface activity, and even the shorter-chain analogues studied here collapsed at low surface pressures. Therefore, it appears unlikely that the naturally occurring ultralong diesters would partition to the aqueous interface instead of the bulk of the nonpolar sublayer. Instead, they may have a role in multilamellar ordering and the organization of the nonpolar lipid layer. The synthesized type I-St diester in particular displayed a tendency to form multilamellar structures, suggesting that diesters of this subtype may be involved in forming the multilamellar lipid structures that have been proposed to exist in the TFLL.^54,55^

In addition to the biophysical properties of the OAHFAs and diesters described here, their interactions with other tear film lipid components, such as wax esters, cholesteryl esters, and phospholipids, is likely essential to the overall function of the tear film. Our study provides a solid and hitherto missing foundation for addressing the role of these lipid classes in the organization and function of the TFLL in addition to multiple new tools that are applicable to ocular surface research and beyond.

## 4 Experimental Section

All reagents were purchased from commercial sources. Dry solvents were purified by the VAC vacuum solvent purification system prior to use when dry solvents were needed. All reactions containing moisture- or air-sensitive reagents were carried out under an argon atmosphere. All reactions requiring heating were performed using an oil bath. TLC was performed on aluminum sheets precoated with silica gel 60 F254 (Merck). Flash chromatography was carried out using silica gel 40. Spots were visualized by UV, then sprayed with a 1:4 H_2_SO_4_/MeOH solution and heated. HRMS spectra were recorded using a Bruker Micro Q-TOF with ESI (electrospray ionization) operated in the positive mode. NMR spectra were recorded with a Bruker Avance III NMR spectrometer operating at 500.13 or 499.82 (^1^H), 125.68 (^13^C,) and 202.40 MHz (^31^P). All products were characterized by a combination of 1D (^1^H, ^13^C{^1^H}, and ^31^P) and 2D techniques (DQF-COSY, TOCSY, Ed-HSQC, and HMBC) with pulse sequences provided by the instrument manufacturer. The probe temperature was kept at 25 °C unless otherwise stated. The chemical shifts are expressed on the δ scale (ppm) using TMS (tetramethylsilane) or residual chloroform as the internal standards. The coupling constants are given in Hertz and provided only once when first encountered. The coupling patterns are given as s (singlet), d (doublet), t (triplet), or m (multiplet). Details on the numbering of the molecules are provided in the Supporting Information. The computational analysis of the ^1^H NMR spectra of all compounds was achieved using of PERCH (PEak reseaRCH) NMR software, and starting values and spectral parameters were obtained from the various NMR techniques used.^56^ Melting point analysis was performed using a Büchi B-545 melting point instrument (BÜCHI Labortechnik AG) when possible.

### 4.1 Synthetic Protocols and Substrate Specific Characterization Data

#### 4.1.1 12-Bromo-1-dodecanol

To a solution containing 1,12-dodecanediol (2 g, 1 equiv) in cyclohexane (26.2 mL) was added HBr (26.2 mL, 23.6 equiv, 48% sol. in H_2_O), and the biphasic system was refluxed for 18 h. The reaction mixture was then cooled to rt, and the organic layer was separated. The aqueous phase was extracted with CH_2_Cl_2_ (5 × 25 mL). The combined organic phase was washed with a saturated aq NaHCO_3_ solution (5 × 25 mL) and brine (50 mL), dried over Na_2_SO_4_, filtered, and concentrated. The crude product was purified by column chromatography using hexane/EtOAc (7:3) and dried on the vacuum line to give the title compound as a white solid (2.03 g, 77% yield): *R*_f_ = 0.34 (hexane/EtOAc 7:3). ^1^H NMR (500.13 MHz, CDCl_3_, 25 °C): δ 3.64 (t, 2H, *J*_1,2_ = 6.5 Hz, H-1), 3.41 (t, 2H, *J*_12,11_= 6.9 Hz, H-12), 1.85 (tt, 2H, *J*_11,10_ = 7.5 Hz, H-11), 1.56 (tt, 2H, *J*_2,3_ = 7.5 Hz, H-2), 1.42 (tt, 2H, *J*_10,9_ = 6.4 Hz, H-10), and 1.38–1.22 (m, 14H, H-3–H-9). ^13^C{^1^H} NMR (125.68 MHz, CDCl_3_, 25 °C): δ 63.2 (C-1), 34.2 (C-12), 33.0 (C-11, C-2), 29.7–28.9 (C-4–C-9), 28.3 (C-10), and 25.9 (C-3). HRMS (EI): *m*/*z* calculated for C_12_H_25_BrONa [M + Na]^+^ 287.0989, found 287.1001.

#### 4.1.2 12-Bromo-1-*tert*-butyldimethylsilyloxydodecane (**1**)

To a solution containing 12-bromo-1-dodecanol (0.500 g, 1.2 equiv) in dry CH_2_Cl_2_ (3 mL) was added imidazole (0.215 g, 2 equiv). The reaction mixture was stirred under an argon atmosphere, and TBDSMCl (0.238 g, 1 equiv) was added after complete dissolution. The reaction mixture was stirred at rt for 18 h, then poured onto a cold saturated aq. NaHCO_3_ solution (20 mL) and extracted with CH_2_Cl_2_ (3 × 20 mL). The combined organic phase was washed with H_2_O (50 mL), dried over Na_2_SO_4_, filtered, and concentrated. The crude oil was purified by column chromatography using hexane/EtOAc (97:3) as the eluent and dried on the vacuum line to give the title compound as a thick colorless oil (0.564 g, 94% yield): *R*_f_= 0.81 (hexane/EtOAc 95:5). ^1^H NMR (500.13 MHz, CDCl_3_, 25 °C): δ 3.60 (t, 2H, *J*_1,2_ = 6.6 Hz, H-1), 3.40 (t, 2H, *J*_12,11_ = 6.9 Hz, H-12), 1.85 (tt, 2H, *J*_11,10_ = 7.5 Hz, H-11), 1.50 (tt, 2H, *J*_1,2_ = 6.6 Hz, H-2), 1.42 (tt, 2H, *J*_10,9_ = 6.6 Hz, H-10), 1.35–1.22 (m, 14H, H-3–H-9), 0.89 (s, 9H, 1-OSi(CH_2_)_2_C(C**H**_3_)_3_), and 0.04 (s, 6H, 1-OSi(C**H**_**3**_)_2_C(CH_3_)_3_). ^13^C{^1^H} NMR (125.68 MHz, CDCl_3_, 25 °C): δ 63.5 (C-1), 34.1 (C-12), 33.0 (C-11, C-2), 29.8–28.9 (C-4–C-9), 28.3 (C-10), 26.1 (1-OSi(CH_3_)_2_C(**C**H_3_)_3_), 26.0 (C-3), 18.5 (1-OSi(CH_3_)_2_**C**(CH_3_)_3_), and −5.1 (1-OSi(**C**H_3_)_2_C(CH_3_)_3_). HRMS (EI): *m*/*z* calculated for C_18_H_39_BrOSiNa [M + Na]^+^ 401.1854, found 401.1852.

#### 4.1.3 1-*tert*-butyldimethylsilyloxydodecane, Triphenylphosphonium Bromide

A mixture containing **1** (2.0 g, 1 equiv) and PPh_3_ (1.39 g, 1 equiv) was stirred under an argon atmosphere at 120 °C o/n and cooled to rt. The formation of the triphenylphosphonium bromide salt was confirmed by ^31^P NMR analysis, and the thick resinous product was used in the subsequent Wittig reaction as such.^31^P NMR (202.4 MHz, CDCl_3_, 25 °C): δ 24.4 (Ph_3_P^+^).

#### 4.1.4 8-Bromo-1-octanal

8-Bromo-1-octanol (1.01 g, 1 equiv) was dissolved in CH_2_Cl_2_ (80 mL), and PCC (1.563 g, 1.5 equiv) was added. The reaction mixture was stirred at rt for 3 h and then Et_2_O (80 mL) was added, followed by filtration through Celite to remove the remnants of PCC. The flask was washed with Et_2_O (2 × 80 mL), and the combined filtrates were filtered through Celite before concentration. H_2_O (50 mL) and Et_2_O (50 mL) were added to this residue. The light green organic phase was separated, and the aqueous phase was extracted with CH_2_Cl_2_ (2 × 50 mL). The combined organic phase was washed with H_2_O (100 mL), dried over Na_2_SO_4_, filtered, and concentrated to give the title compound as an oil (0.84 g, 84% yield). The crude product was used in the subsequent Wittig reaction as such. ^1^H NMR (500.13 MHz, CDCl_3_, 25 °C): δ 9.74 (t, 1H, *J*_1,2_ = 1.6 Hz, H-1), 3.38 (t, 2H, *J*_8,7_ = 6.8 Hz, H-8), 2.41 (dt, 2H, *J*_2,3_ = 7.3 Hz, H-2), 1.83 (tt, 2H, *J*_7,6_ = 7.0 Hz, H-7), 1.61 (tt, 2H, *J*_3,4_ = 7.4 Hz, H-3), 1.42 (tt, 2H, *J*_6,5_ = 7.0 Hz, H-6), and 1.35–1.29 (m, 4H, H-4, H-5).

#### 4.1.5 (12*Z*)-20-Bromo-1-*tert*-butyldimethylsilyloxyeicos-12-ene (**2**)

A solution containing 1-*tert*-butyldimethylsilyloxydodecane, triphenylphosphonium bromide (3.164 g, 2 equiv) in dry THF (30 mL), and HMPA (8.9 mL) under an argon atmosphere was cooled to −78 °C. After 10 min, NaHMDS (8.2 mL, 0.6 M in toluene, 2 equiv) was slowly added, and the resulting mixture was stirred for 1 h. A solution of freshly prepared 8-bromo-1-octanal (0.536 g, 1.05 equiv) dissolved in dry THF (8 mL) was slowly added at −78 °C, and the reaction mixture was allowed to warm to rt over 24 h before it was quenched with an aq phosphate buffer (freshly prepared, pH 7.2, 80 mL). Extraction with Et_2_O (3 × 80 mL) was performed, and the combined organic phase was dried over Na_2_SO_4_, filtered, and concentrated. The crude product was purified by column chromatography using a hexane/Et_3_N (100:0.1) to hexane/EtOAc/Et_3_N (399:1:0.1 → 98.7:1.3:0.1) gradient as the eluent and further dried on the vacuum line to give the title compound as a thick yellowish oil (0.427 g, 34% yield): *R*_f_ = 0.31 (hexane/EtOAc/Et_3_N 98:1:1). ^1^H NMR (499.82 MHz, CDCl_3_, 25 °C): δ 5.35 (dtt, 1H, *J*_12,14_ = −1.5, *J*_12,11_ = 7.1, *J*_12,13_ = 11.0 Hz, H-12), 5.34 (dtt, 1H, *J*_13,11_ = −1.5, *J*_13,14_ = 7.0 Hz, H-13), 3.60 (t, 2H, *J*_1,2_ = 6.6 Hz, H-1), 3.40 (t, 2H, *J*_20,19_ = 6.9 Hz, H-20), 2.02 (ddt, 2H, *J*_14,15_ = 6.7 Hz, H-14), 2.01 (ddt, 2H, *J*_11,10_ = 7.4 Hz, H-11), 1.85 (tt, 2H, *J*_19,18_ = 7.5 Hz, H-19), 1.50 (tt, 2H, *J*_2,3_ = 6.7 Hz, H-2), 1.43 (tt, 2H, *J*_18,17_ = 6.7 Hz, H-18), 1.38–1.22 (m, 22H, H-3–H-10, H-15–H-17), 0.89 (s, 9H, 1-OSi(CH_2_)_2_C(C**H**_**3**_)_3_), and 0.05 (s, 6H, 1-OSi(C**H**_**3**_)_2_C(CH_3_)_3_). ^13^C{^1^H} NMR (125.68 MHz, CDCl_3_, 25 °C): δ 130.3 (C-12), 129.9 (C-13), 63.5 (C-1), 34.2 (C-20), 33.1 (C-2), 33.0 (C-19), 29.9–28.8 (C-4–C-10, C-15–C-17), 28.3 (C-18), 27.4–27.3 (C-11, C-14), 26.2 (1-OSi(CH_3_)_2_C(**C**H_3_)_3_), 26.0 (C-3), 18.6 (1-OSi(CH_3_)_2_**C**(CH_3_)_3_), and −5.1 (1-OSi(**C**H_3_)_2_ C(CH_3_)_3_). HRMS (EI): *m*/*z* calculated for C_26_H_53_BrOSiNa [M + Na]^+^ 511.2949, found 511.2995.

#### 4.1.6 (12*Z*)-20-Acetoxy-1-*tert*-butyldimethylsilyloxyeicos-12-ene

To a solution containing **2** (0.265 g, 1 equiv) in DMSO (12 mL) was added KOAc (0.266 g, 5 equiv), and the suspension was stirred at rt o/n. After 24 h, additional KOAc (0.159 g, 3 equiv) was added, and the temperature was raised to 50 °C. After 27 h, the reaction mixture was brought to rt, and H_2_O (25 mL) was added. Extraction with Et_2_O (3 × 25 mL) was performed, and the combined organic phase was washed with brine (30 mL), dried over Na_2_SO_4_, filtered, and concentrated. The crude product was purified by column chromatography using hexane/EtOAc/Et_3_N (98:2:0.1 → 95.5:0.1) as the eluent and dried on the vacuum line to give the title compound as a yellowish oil (0.169 g, 66% yield): *R*_f_ = 0.51 (hexane/EtOAc/Et_3_N 95:5:0.1). ^1^H NMR (500.13 MHz, CDCl_3_, 25 °C): δ 5.35 (dtt, 1H, *J*_12,14_ = −1.5, *J*_12,11_ = 7.1, *J*_12,13_ = 11.1 Hz, H-12), 5.34 (dtt, 1H, *J*_13,11_ = −1.5, *J*_13,14_ = 7.2 Hz, H-13), 4.05 (t, 2H, *J*_20,19_ = 6.8 Hz, H-20), 3.59 (t, 2H, *J*_1,2_ = 6.7 Hz, H-1), 2.04 (s, 3H, 20–OCOC**H**_**3**_), 2.01 (ddt, 2H, *J*_14,15_ = 6.0 Hz, H-14), 2.00 (ddt, 2H, *J*_11,10_ = 7.0 Hz, H-11), 1.62 (tt, 2H, *J*_19,18_ = 7.0 Hz, H-19), 1.50 (tt, 2H, *J*_2,3_ = 6.6 Hz, H-2), 1.39–1.22 (m, 24H, H-3–H-10, H-15–H-18), 0.89 (s, 9H, 1-OSi(CH_2_)_2_C(C**H**_**3**_)_3_), and 0.04 (s, 6H, 1-OSi(C**H**_**3**_)_2_C(CH_3_)_3_). ^13^C{^1^H} NMR (125.68 MHz, CDCl_3_, 25 °C): δ 171.4 (20-O**C**OCH_3_), 130.2 (C-12), 129.9 (C-13), 64.8 (C-20), 63.5 (C-1), 33.1 (C-2), 29.9–29.3 (C-4–C-10, C-15–C-17), 28.8 (C-19), 27.4–27.3 (C-11, C-14), 26.2 (1-OSi(CH_3_)_2_C(**C**H_3_)_3_), 26.0 (C-3, C-18), 21.2 (20–OCO**C**H_3_), 18.6 (1-OSi(CH_3_)_2_**C**(CH_3_)_3_), and −5.1 (1-OSi(**C**H_3_)_2_C(CH_3_)_3_). HRMS (EI): *m*/*z* calculated for C_28_H_56_O_3_SiNa (M + Na^+^) 491.3899, found 491.3879.

#### 4.1.7 (12*Z*)-20-Hydroxy-1-*tert*-butyldimethylsilyloxyeicos-12-ene (**3**)

To a solution containing (12*Z*)-20-acetoxy-1-*tert*-butyldimethylsilyloxyeicos-12-ene (0.022 g, 1 equiv) in MeOH (1 mL) and THF (0.5 mL) under argon atmosphere was added NaOMe (0.003 g, 1 equiv), and the resulting mixture was stirred at rt. After 22 h, the reaction was quenched by the addition of aq HCl (10% sol. v/v) and H_2_O (15 mL). The resulting mixture was extracted with Et_2_O (3 × 15 mL), and the combined organic phase was dried over Na_2_SO_4_, filtered, and concentrated. Drying under vacuum gave the title compound as a white solid (0.015 g, 78% yield): *R*_f_ = 0.56 (hexane/EtOAc/Et_3_N 7:3:0.1). ^1^H NMR (500.13 MHz, CDCl_3_, 25 °C): δ 5.34 (dtt, 1H, *J*_12,14_ = −1.0, *J*_12,11_ = 7.0, *J*_12,13_ = 11.3 Hz, H-12), 5.34 (dtt, 1H, *J*_13,11_ = −1.0, *J*_13,14_ = 7.0 Hz, H-13), 3.64 (t, 2H, *J*_1,2_ = 6.7 Hz, H-1), 3.59 (t, 2H, *J*_20,19_ = 6.7 Hz, H-20), 2.01 (ddt, 2H, *J*_14,15_ = 6.3 Hz, H-14), 2.00 (ddt, 2H, *J*_11,10_ = 7.4 Hz, H-11), 1.57 (tt, 2H, *J*_2,3_ = 7.4 Hz, H-2), 1.50 (tt, 2H, *J*_19,18_ = 6.6 Hz, H-19), 1.40–1.22 (m, 24H, H-3–H-10, H-15–H-18), 0.89 (s, 9H, 1-OSi(CH_2_)_2_C(C**H**_**3**_)_3_), and 0.04 (s, 6H, 1-OSi(C**H**_**3**_)_2_C(CH_3_)_3_). ^13^C{^1^H} NMR (125.68 MHz, CDCl_3_, 25 °C): δ 130.2 (C-12), 129.9 (C-13), 63.5 (C-20), 63.3 (C-1), 33.0 (C-19), 32.9 (C-2), 29.9–29.4 (C-4–C-10, C-15–C-17), 27.4–27.3 (C-11, C-14), 26.1 (1-OSi(CH_3_)_2_C(**C**H_3_)_3_), 26.0–25.9 (C-3, C-18), 18.6 (1-OSi(CH_3_)_2_**C**(CH_3_)_3_), and −5.1 (1-OSi(**C**H_3_)_2_C(CH_3_)_3_). HRMS (EI): *m*/*z* calculated for C_26_H_54_O_2_SiNa [M + Na]^+^ 449.3793, found 449.3832.

#### 4.1.8 (12*Z*)-20-Oleoyloxy-1-*tert*-butyldimethylsilyloxyeicos-12-ene

To a solution of **3** (0.040 g, 1 equiv) in dry CH_2_Cl_2_ (2 mL) under an argon atmosphere were added DMAP (0.012 g, 1 equiv) and EDC·HCl (0.046 g, 2.5 equiv), and the resulting mixture was cooled to 0 °C on an ice-bath. Oleic acid (0.032 g dissolved in 0.5 mL of dry CH_2_Cl_2_, 1.2 equiv) was added, and the reaction mixture was stirred at 0 °C for 10 min and then at rt o/n. The reaction mixture was quenched after 20 h with H_2_O (2 mL) and diluted with CH_2_Cl_2_ (10 mL). The organic phase was separated, and the aqueous phase was extracted with CH_2_Cl_2_ (2 × 10 mL). The combined organic phase was washed with H_2_O (2 × 15 mL), dried over Na_2_SO_4_, filtered, and concentrated. The crude product was purified by column chromatography using hexane/EtOAc/Et_3_N (95:5:0.1) as the eluent and dried under vacuum to give the title compound as a white solid (0.061 g, 93% yield): *R*_f_ = 0.46 (hexane/EtOAc/Et_3_N 95:5:0.1). ^1^H NMR (500.13 MHz, CDCl_3_, 25 °C): δ 5.35 (dtt, 1H, *J*_9′,11′_ = −1.1, *J*_9′,8′_ = 7.1, *J*_9′,10′_ = 11.6 Hz, H-9′), 5.35 (dtt, 1H, *J*_12,14_ = −1.2, *J*_12,11_ = 7.1, *J*_12,13_ = 10.5 Hz, H-12), 5.34 (dtt, 1H, *J*_10′,8′_ = −1.2, *J*_10′,11′_ = 6.8 Hz, H-10′), 5.34 (dtt, 1H, *J*_13,11_ = −1.5, *J*_13,14_ = 6.6 Hz, H-13), 4.05 (t, 2H, *J*_20,19_ = 6.7 Hz, H-20), 3.60 (t, 2H, *J*_1,2_ = 6.7 Hz, H-1), 2.29 (t, 2H, *J*_2′,3′_ = 7.3 Hz, H-2′), 2.06–1.96 (m, 8H, H-11, H-14, H-8′, H-11′), 1.62 (tt, 2H, *J*_3′,4′_ = 6.7 Hz, H-3′), 1.61 (tt, 2H, *J*_19,18_ = 6.6 Hz, H-19), 1.50 (tt, 2H, *J*_2,3_ = 6.7 Hz, H-2), 1.37–1.23 (m, 44H, H-3–H-10, H-15–H-18, H-4′–H-7′, H-12′–H-17′), 0.89 (s, 9H, 1-OSi(CH_2_)_2_C(C**H**_**3**_)_3_), 0.88 (t, 3H, *J*_18′,17′_ = 7.1 Hz, H-18′), and 0.05 (s, 6H, 1-OSi(C**H**_**3**_)_2_C(CH_3_)_3_). ^13^C{^1^H} NMR (125.68 MHz, CDCl_3_, 25 °C): δ 174.1 (C-1′), 130.2–129.9 (C-12, C-13, C-9′, C-10′), 64.5 (C-20), 63.5 (C-1), 34.5 (C-2′), 33.0 (C-2), 32.1 (C-16′), 29.9–29.3 (C-4–C-10, C-15–C-17, C-4′–C-7′, C-12′–C-15′), 28.8 (C-19), 27.4–27.3 (C-11, C-14, C-8′, C-11′), 26.1 (1-OSi(CH_2_)_2_C(**C**H_3_)_3_), 26.0 (C-3, C-18), 25.2 (C-3′), 22.8 (C-17′),18.6 (1-OSi(CH_3_)_2_**C**(CH_3_)_3_), 14.3 (C-18′), and −5.1 (1-OSi(**C**H_3_)_2_C(CH_3_)_3_). HRMS (EI): *m*/*z* calculated for C_46_H_86_O_3_SiNa [M + Na]^+^ 713.6246, found 713.6214.

#### 4.1.9 (12*Z*)-20-Oleoyloxyeicos-12-enol (**4**)

A solution containing (12*Z*)-20-oleoyloxy-1-*tert*-butyldimethylsilyloxyeicos-12-ene (0.031 g, 1 equiv) in dry THF (0.5 mL) under an argon atmosphere was cooled to 0 °C on an ice-bath, and TBAF (0.140 mL, 1 M in THF, 3 equiv) was added. After 5 min, the ice-bath was removed, and the reaction mixture was stirred at rt for 1 h before being quenched with H_2_O (2 mL) and extracted with EtOAc (3 mL). The organic phase was washed with H_2_O (2 × 5 mL), and the aqueous phase was re-extracted with EtOAc (2 × 5 mL). The combined organic phase was dried over Na_2_SO_4_, filtered, and concentrated. The crude product was purified by column chromatography using hexane/EtOAc/Et_3_N (7:3:0.1) as the eluent and dried under vacuum to give the title compound as a white solid (0.023 g, 92% yield): *R*_f_ = 0.58 (hexane/EtOAc/Et_3_N 7:3:0.1). ^1^H NMR (500.13 MHz, CDCl_3_, 25 °C): δ 5.35 (dtt, 1H, *J*_9′,11′_ = −2.1, *J*_9′,8′_ = 6.4, *J*_9′,10′_ = 10.9 Hz, H-9′), 5.35 (dtt, 1H, *J*_12,14_ = −1.8, *J*_12,11_ = 7.3, *J*_12,13_ = 11.5 Hz, H-12), 5.34 (dtt, 1H, *J*_10′,8′_ = −1.0, *J*_10′,11′_ = 7.2 Hz, H-10′), 5.34 (dtt, 1H, *J*_13,11_ = −2.0, *J*_13,14_ = 6.6 Hz, H-13), 4.05 (t, 2H, *J*_20,19_ = 6.7 Hz, H-20), 3.64 (t, 2H, *J*_1,2_ = 6.6 Hz, H-1), 2.29 (t, 2H, *J*_2′,3′_ = 7.3 Hz, H-2′), 2.06–1.96 (m, 8H, H-11, H-14, H-8′, H-11′), 1.62 (tt, 2H, *J*_3′,4′_ = 6.7 Hz, H-3′), 1.61 (tt, 2H, *J*_19,18_ = 6.6 Hz, H-19), 1.56 (tt, 2H, *J*_2,3_ = 6.7 Hz, H-2), 1.37–1.23 (m, 44H, H-3–H-10, H-15–H-18, H-4′–H-7′, H-12′–H-17′), and 0.88 (t, 3H, *J*_18′,17′_ = 7.1 Hz, H-18′). ^13^C{^1^H} NMR (125.68 MHz, CDCl_3_, 25 °C): δ 174.2 (C-1′), 130.2–129.9 (C-12, C-13, C-9′, C-10′), 64.5 (C-20), 63.2 (C-1), 34.5 (C-2′), 33.0 (C-2), 32.1 (C-16′), 29.9–29.3 (C-4–C-10, C-15–C-17, C-4′–C-7′, C-12′–C-15′), 28.8 (C-19), 27.4–27.3 (C-11, C-14, C-8′, C-11′), 26.0–25.9 (C-3, C-18), 25.2 (C-3′), 22.8 (C-17′), and 14.3 (C-18′). HRMS (EI): *m*/*z* calculated for C_38_H_72_O_3_Na [M + Na]^+^ 599.5381, found 599.5342.

#### 4.1.10 (12*Z*)-20-Oleoyloxyeicos-12-enoic acid (**5**)

A solution containing **4** (0.026 g, 1 equiv) in acetone (2 mL) and EtOAc (2 mL) was cooled to 0 °C on an ice-bath, and the Jones reagent (0.050 mL, 2.2 equiv) was added. The resulting mixture was stirred at 0 °C for 45 min. H_2_O (5 mL) was added ,and the reaction mixture was then extracted with Et_2_O (3 × 15 mL). The combined organic phase was washed with brine (15 mL), dried over Na_2_SO_4_, filtered, and concentrated. The crude product was purified by column chromatography using hexane/EtOAc/AcOH (7:3:0.1) as the eluent and dried on the vacuum line to give the title compound as a white solid (0.024 g, 89% yield): *R*_f_ = 0.43 (hexane/EtOAc/AcOH 7:3:0.1). ^1^H NMR (500.13 MHz, CDCl_3_, 25 °C): δ 5.35 (dtt, 1H, *J*_9′,11′_ = −1.3, *J*_9′,8′_ = 6.9, *J*_9′,10′_ = 11.1 Hz, H-9′), 5.35 (dtt, 1H, *J*_12,14_ = −1.8, *J*_12,11_ = 7.3, *J*_12,13_ = 11.6 Hz, H-12), 5.34 (dtt, 1H, *J*_10′,8′_ = −0.8, *J*_10′,11′_ = 7.2 Hz, H-10′), 5.34 (dtt, 1H, *J*_13,11_ = −2.2, *J*_13,14_ = 6.8 Hz, H-13), 4.05 (t, 2H, *J*_20,19_ = 6.8 Hz, H-20), 2.34 (t, 2H, *J*_2,3_ = 7.1 Hz, H-2), 2.29 (t, 2H, *J*_2′,3′_ = 7.3 Hz, H-2′), 2.06–1.96 (m, 8H, H-11, H-14, H-8′, H-11′), 1.63 (tt, 2H, *J*_3,4_ = 6.4 Hz, H-3), 1.61 (tt, 2H, *J*_3′,4′_ = 6.6 Hz, H-3′), 1.61 (tt, 2H, *J*_19,18_ = 6.6 Hz, H-19), 1.37–1.23 (m, 42H, H-4–H-10, H-15–H-18, H-4′–H-7′, H-12′–H-17′), and 0.88 (t, 3H, *J*_18′,17′_ = 7.1 Hz, H-18′). ^13^C{^1^H} NMR (125.68 MHz, CDCl_3_, 25 °C): δ 179.6 (C-1), 174.2 (C-1′), 130.1–129.9 (C-12, C-13, C-9′, C-10′), 64.6 (C-20), 34.5 (C-2′), 34.4 (C-2), 32.0 (C-16′), 29.9–29.3 (C-4–C-10, C-15–C-17, C-4′–C-7′, C-12′–C-15′), 28.8 (C-19), 27.4–27.3 (C-11, C-14, C-8′, C-11′), 26.1 (C-18), 25.2 (C-3′), 24.9 (C-3), 22.8 (C-17′), and 14.3 (C-18′). HRMS (EI): *m*/*z* calculated for C_38_H_70_O_4_Na [M + Na]^+^ 613.5174, found 613.5175. mp: 29.5–30.7 °C.

#### 4.1.11 Cholesteryl-(12*Z*)-20-oleoyloxyeicos-12-enoate (**6**)

Cholesterol (0.019 g, 1.2 equiv) was dissolved in dry CH_2_Cl_2_ (1.5 mL) under an argon atmosphere, then DMAP (0.005 g, 1 equiv) and EDC·HCl (0.020 g, 2.5 equiv) were added. The reaction mixture was cooled on an ice bath and then **5** (0.024 g in 1 mL of dry CH_2_Cl_2_; 1 equiv) was added. The resulting mixture was stirred at 0 °C for 10 min and then at rt o/n. The reaction mixture was quenched after 20 h with H_2_O (5 mL) and diluted with CH_2_Cl_2_ (10 mL). The organic phase was separated, and the aqueous phase was extracted with CH_2_Cl_2_ (2 × 10 mL). The combined organic layers were washed with H_2_O (2 × 15 mL), dried over Na_2_SO_4_, filtered, and concentrated. The crude product was purified by column chromatography using hexane/EtOAc/Et_3_N (100:0:0.1–95:5:0.1) as the eluent and dried on the vacuum line to give the title compound as a yellow oil (0.026 g, 67% yield): *R*_f_ = 0.27 (hexane/EtOAc/Et_3_N 95:5:0.1). ^1^H NMR (499.82 MHz, CDCl_3_): δ 5.37 (dddd, 1H, *J*_6,4a_ = −0.3, *J*_6,4b_ = −1.9, *J*_6,7a_ = 2.0, *J*_6,7b_ = 5.2 Hz, H-6), 5.35 (dtt, 1H, *J*_9″,11′′_ = −1.1, *J*_9″,8′′_ = 6.7, *J*_9″,10′′_ = 11.3 Hz, H-9″), 5.35 (dtt, 1H, *J*_12′,14′_ = −1.5, *J*_12′,11′_ = 6.7, *J*_12′,13′_ = 11.6 Hz, H-12′), 5.34 (dtt, ^1^H, *J*_10″,8′′_ = −1.5, *J*_10″,11′′_ = 7.2 Hz, H-10″), 5.34 (dtt, 1H, *J*_13′,11′_ = −2.0, *J*_13′,14′_ = 6.8 Hz, H-13′), 4.61 (dddd, 1H, *J*_3,2a_ = 4.7, *J*_3,4a_ = 4.8, *J*_3,4b_ = 11.3, *J*_3,2b_ = 11.4 Hz, H-3), 4.05 (t, 2H, *J*_20′,19′_ = 6.7 Hz, H-20′), 2.31 (dddd, 1H, *J*_4a,2a_ = −2.5, *J*_4a,4b_ = −13.0 Hz, H-4a), 2.30 (ddddd, 1H, *J*_4b,7b_ = −2.7, *J*_4b,7a_ = −3.3 Hz, H-4b), 2.29 (t, 2H, *J*_2′,3′_ = 7.7 Hz, H-2′), 2.26 (t, 2H, *J*_2″,3′′_ = 7.6 Hz, H-2″), 2.05–1.98 (m, 9H, H-12a, H-11′, H-14′, H-8′′, H-11′′), 1.97 (dddd, 1H, *J*_7a,8_ = 4.9, *J*_7a,7b_ = −17.6 Hz, H-7a), 1.85 (ddd, 1H, *J*_1a,2b_ = 3.3, *J*_1a,2a_ = 3.4, *J*_1a,1b_ = −13.6 Hz, H-1a), 1.84 (dddd, 1H, *J*_2a,1b_ = 3.7, *J*_2a,2b_ = −12.2 Hz, H-2a), 1.82 (dddd, 1H, *J*_16a,15b_ = 6.0, *J*_16a,17_ = 9.1, *J*_16a,15a_ = 9.6, *J*_16a,16b_ = −13.4 Hz, H-16a), 1.65–1.55 (m, 8H, H-3′, H-19′, H-3″, H-2b, H-15a), 1.55–1.45 (m, 4H, H-7b, H-25, H-11a, H-11b), 1.44 (dddd, 1H, *J*_8,14_ = 10.3, *J*_8,9_ = 11.7 Hz, H-8), 1.40–1.20 (m, 46H, H-4′–H-10′, H-15′–H-18′, H-4″–H-7′′, H-12′′–H-17′′, H-16b, H-20, H-22a, H-23a), 1.16 (ddd, 1H, *J*_12b,11a_ = 3.2, *J*_12b,12a_ = −12.8, *J*_12b,11b_ = 13.9 Hz, H-12b), 1.15–1.04 (m, 6H, H-1b, H-15b, H-17, H-23b, H-24a, H-24b), 1.02 (s, 3H, H-19), 1.00 (dddd, 1H, *J*_22b,23a_ = 4.8, *J*_22b,23b_ = 9.0, *J*_22b,20_ = 9.0, *J*_22b,22a_ = −13.6 Hz, H-22b), 0.99 (ddd, 1H, *J*_14,15a_ = 7.6, *J*_14,15b_ = 12.6 Hz, H-14), 0.95 (ddd, 1H, *J*_9,11a_ = 3.8, *J*_9,11b_ = 12.3 Hz, H-9), 0.91 (d, 3H, H-21), 0.88 (t, 3H, *J*_18″,17′′_ = 6.8 Hz, H-18″), 0.87, 0.86 (each d, each 3H, each *J*_26/27,25_ = 6.6 Hz, H-26, and H-27), and 0.67 (s, 3H, H-18). ^13^C{^1^H} NMR (125.68 MHz, CDCl_3_): δ 174.1(C-1′), 173.5 (C-1″), 139.9 (C-5), 130.2–129.9 (C-9′′, C-10′′, C-12′, C-13′), 122.7 (C-6), 73.8 (C-3), 64.5 (C-20′), 56.8 (C-14), 56.3 (C-17), 50.2 (C-9), 42.5 (C-13), 39.9 (C-12), 39.7 (C-24), 38.3 (C-4), 37.2 (C-1), 36.8 (C-10), 36.3 (C-22), 35.9 (C-20), 34.9 (C-2′′), 34.6 (C-2′), 32.1–32.0 (C-7, C-8, C-16′′), 29.9–29.3 (C-4′–C-10′, C-15′–C-17′, C-4′′–C-7′′, C-12′′–C-15′′), 28.8 (C-19′), 28.4 (C-16), 28.2 (C-25), 28.0 (C-2), 27.4–27.3 (C-11′, C-14′, C-8′′, C-11′′), 26.0 (C-18′), 25.2–25.1 (C-3′, C-3′′), 24.4 (C-15), 24.0 (C-23), 23.0 and 22.7 (C-26, C-27), 22.8 (C-17′′), 21.2 (C-11), 19.5 (C-19), 18.9 (C-21), 14.3 (C-18′′) and 12.0 (C-18). HRMS (EI): *m*/*z* calculated for C_65_H_114_O_4_Na [M + Na]^+^ 981.8617, found 981.8614.

#### 4.1.12 (12*Z*)-1,20-dioleoyloxyeicos-12-ene (**7**)

To a solution of **4** (0.031 g, 1 equiv) in dry CH_2_Cl_2_ (1.5 mL) under an argon atmosphere were added DMAP (0.007 g, 1 equiv) and EDC·HCl (0.027 g, 2.5 equiv). The reaction mixture was cooled on an ice bath, and oleic acid (0.019 g in 0.5 mL dry CH_2_Cl_2_; 1.2 equiv) was added. The resulting mixture was stirred for 10 min at 0 °C and then at rt o/n. The reaction mixture was quenched after 20 h with H_2_O (2 mL) and diluted with CH_2_Cl_2_ (10 mL). The organic phase was separated, and the aqueous layer was extracted with CH_2_Cl_2_ (2 × 10 mL). The combined organic layers were washed with H_2_O (2 × 15 mL), dried over Na_2_SO_4_, filtered, and concentrated. The crude product was purified by column chromatography using hexane/EtOAc/Et_3_N (95:5:0.1) as the eluent and dried on the vacuum line to give the title compound as an oil (0.032 g, 70% yield): *R*_f_ = 0.59 (hexane/EtOAc/Et_3_N 95:5:0.1). ^1^H NMR (499.82 MHz, CDCl_3_, 25 °C): δ 5.39–5.30 (m, 6H, H-9, H-10, H-12′, H-13′, H-9″, H-10′′), 4.05 (t, 2H, *J*_1′,2′_ = 6.7 Hz, H-1′), 4.05 (t, 2H, *J*_20′,19′_ = 6.7 Hz, H-20′), 2.28 (t, 2H, *J*_2,3_ = 7.5 Hz, H-2), 2.28 (t, 2H, *J*_2″,3′′_ = 7.5 Hz, H-2″), 2.05–1.96 (m, 12H, H-8, H-11, H-11′, H-14′, H-8′′, H-11′′), 1.61 (tt, 2H, *J*_3,4_ = 6.7 Hz, H-3), 1.61 (tt, 2H, *J*_2′,3′_ = 7.0 Hz, H-2′), 1.61 (tt, 2 H, *J*_19′,18′_ = 7.1 Hz, H-19′), 1.61 (tt, 2H, *J*_3″,4′′_ = 6.6 Hz, H-3″), 1.37–1.20 (m, 64H, H-4–H-7, H-12–H-17, H-4′–H-10′, H-15′–H-18′, H-4′′–H-7′′, H-12′′–H-17′′), 0.88 (t, 3H, *J*_18,17_ = 7.1 Hz, H-18), and 0.88 (t, 3H, *J*_18″,17′′_ = 7.1 Hz, H-18″). ^13^C{^1^H} NMR (125.68 MHz, CDCl_3_, 25 °C): δ 174.1 (C-1, C-1″), 130.1–129.9 (C-9′′, C-10′′, C-12′, C-13′, C-9, C-10), 64.5 (C-1′, C-20′), 34.6 (C.2, C-2′′), 32.1 (C-16, C-16′′), 29.9–29.3 (C-4–C-7, C-12–C-15, C-4′–C-10′, C-15′–C-17′, C-4′′–C-7′′, C-12′′–C-15′′), 28.8 (C-2′, C-19′), 27.4–27.3 (C-8, C-11, C-11′, C-14′, C-8′′, C-11′′), 26.1 (C-3′, C-18′), 25.2 (C-3, C-3′′), 22.8 (C-17, C-17′′), and 14.3 (C-18, C-18′′). HRMS (EI): *m*/*z* calculated for C_56_H_104_O_4_Na [M + Na]^+^ 863.7835, found 863.7818.

### 4.2 Langmuir Monolayer Experiments

Lipids were spread to the air-buffer interface of a KSV NIMA Langmuir large trough (Biolin Scientific, Espoo, Finland; dimensions 580 × 145 mm) filled with PBS buffer in 5 (20:1-OAHFA), 4.87 (20:1-DiE) and 6.41 mM (20:1-St-DiE) chloroform solutions. The measurement setup was contained in an acrylic enclosure (volume 290 L), and dry air was continuously passed through an ozone solutions ODS-3P ozone destruct unit (Hull, Iowa) and into the enclosure at a rate of 76 L/min to maintain a low-ozone atmosphere and prevent the oxidation of the studied lipids during the measurements. Before starting the measurements, chloroform was allowed to evaporate for 3 min. The films were compressed at a constant rate of 10 mm/min (3.7%/min from the initial area) in measurements with BAM or surface potential and a rate of 60 mm/min (22.2%/min from the initial area) in compression–relaxation cycles (Figure S26). The surface pressure was measured using a Wilhelmy plate, and the surface potential was measured using KSV SPOT (Espoo, Finland). Brewster angle microscopy images were captured using a KSV NIMA microBAM camera (Espoo, Finland). A circulating water bath (LAUDA ECO E4) was used to control the temperature of the subphase. Measurements were performed at room temperature, 35 ± 1, and 40 ± 1 °C for all lipids. All measurements were repeated at least three times to ensure repeatability.

### 4.3 Evaporation Measurements

Evaporation measurements were conducted using a modified Langmuir–Schaefer method^46^ as described previously.^51^ In short, the lipids were spread to the air-buffer interface of a KSV minitrough (Helsinki, Finland), the lipid film was compressed to a desired mean molecular area (3.5–40 Å^2^/molecule), and the evaporation rate through the film was measured by placing a box filled with silica gel above the film surface and measuring the amount of water absorbed by the desiccant. The measurement time was 5 min. Disposable desiccant cartridges (SP Industries, Warminster, PA) with silica gel were used, but the membrane was replaced with a Millipore Immobilon-P PVDF membrane with a 450 nm pore size (Bedford, MA). Evaporation measurements were repeated three times for each lipid.

## Supporting Information Available

Numbering of the molecules studied in this work, NMR spectra of synthesized compounds, and supporting figures related to biophysical experiments (PDF)

## References

[ref1] Willcox M. D. P.; Argüeso P.; Georgiev G. A.; Holopainen J. M.; Laurie G. W.; Millar T. J.; Papas E. B.; Rolland J. P.; Schmidt T. A.; Stahl U.; Suarez T.; Subbaraman L. N.; Uçakhan O. Ö.; Jones L. TFOS DEWS II Tear Film Report. Ocul Surf. 2017, 15 (3), 366–403. 10.1016/j.jtos.2017.03.006. 28736338 PMC6035753

[ref2] Stapleton F.; Alves M.; Bunya V. Y.; Jalbert I.; Lekhanont K.; Malet F.; Na K.-S.; Schaumberg D.; Uchino M.; Vehof J.; Viso E.; Vitale S.; Jones L. TFOS DEWS II Epidemiology Report. Ocul Surf. 2017, 15 (3), 334–365. 10.1016/j.jtos.2017.05.003. 28736337

[ref3] Yu J.; Asche C. V.; Fairchild C. J. The Economic Burden of Dry Eye Disease in the United States: A Decision Tree Analysis. Cornea 2011, 30 (4), 379–387. 10.1097/ICO.0b013e3181f7f363. 21045640

[ref4] McDonald M.; Patel D. A.; Keith M. S.; Snedecor S. J. Economic and Humanistic Burden of Dry Eye Disease in Europe, North America, and Asia: A Systematic Literature Review. Ocul Surf. 2016, 14 (2), 144–167. 10.1016/j.jtos.2015.11.002. 26733111

[ref5] Craig J. P.; Nichols K. K.; Akpek E. K.; Caffery B.; Dua H. S.; Joo C.-K.; Liu Z.; Nelson J. D.; Nichols J. J.; Tsubota K.; Stapleton F. TFOS DEWS II Definition and Classification Report. Ocul Surf. 2017, 15 (3), 276–283. 10.1016/j.jtos.2017.05.008. 28736335

[ref6] Craig J. P.; Tomlinson A. Importance of the Lipid Layer in Human Tear Film Stability and Evaporation. Opt Vis Sci. 1997, 74 (1), 8–13. 10.1097/00006324-199701000-00014. 9148269

[ref7] King-Smith P. E.; Hinel E. A.; Nichols J. J. Application of a Novel Interferometric Method to Investigate the Relation between Lipid Layer Thickness and Tear Film Thinning. Invest. Ophthalmol. Visual Sci. 2010, 51 (5), 2418–2423. 10.1167/iovs.09-4387. 20019370 PMC3259007

[ref8] King-Smith P. E.; Reuter K. S.; Braun R. J.; Nichols J. J.; Nichols K. K. Tear Film Breakup and Structure Studied by Simultaneous Video Recording of Fluorescence and Tear Film Lipid Layer Images. Invest. Ophthalmol. Visual Sci. 2013, 54 (7), 4900 10.1167/iovs.13-11878. 23766476 PMC3720150

[ref9] Dursch T. J.; Li W.; Taraz B.; Lin M. C.; Radke C. J. Tear-Film Evaporation Rate from Simultaneous Ocular-Surface Temperature and Tear-Breakup Area. Opt Vis Sci. 2018, 95 (1), 5–12. 10.1097/OPX.0000000000001156. 29252906

[ref10] Butovich I. A. Tear Film Lipids. Exp. Eye Res. 2013, 117, 4–27. 10.1016/j.exer.2013.05.010. 23769846 PMC3844095

[ref11] Brown S. H. J.; Kunnen C. M. E.; Duchoslav E.; Dolla N. K.; Kelso M. J.; Papas E. B.; Lazon de la Jara P.; Willcox M. D. P.; Blanksby S. J.; Mitchell T. W. A Comparison of Patient Matched Meibum and Tear Lipidomes. Invest. Ophthalmol. Visual Sci. 2013, 54 (12), 7417–7424. 10.1167/iovs.13-12916. 24135754

[ref12] Chen J.; Green K. B.; Nichols K. K. Quantitative Profiling of Major Neutral Lipid Classes in Human Meibum by Direct Infusion Electrospray Ionization Mass Spectrometry. Invest. Ophthalmol. Visual Sci. 2013, 54 (8), 5730–5753. 10.1167/iovs.12-10317. 23847307 PMC3753034

[ref13] Kunnen C. M. E.; Brown S. H. J.; de la Jara P. L.; Holden B. A.; Blanksby S. J.; Mitchell T. W.; Papas E. B. Influence of Meibomian Gland Expression Methods on Human Lipid Analysis Results. Ocul Surf. 2016, 14 (1), 49–55. 10.1016/j.jtos.2015.10.001. 26524238

[ref14] Lam S. M.; Tong L.; Reux B.; Duan X.; Petznick A.; Yong S. S.; Khee C. B. S.; Lear M. J.; Wenk M. R.; Shui G. Lipidomic Analysis of Human Tear Fluid Reveals Structure-Specific Lipid Alterations in Dry Eye Syndrome. J. Lipid Res. 2014, 55 (2), 299–306. 10.1194/jlr.P041780. 24287121 PMC3886668

[ref15] Lam S. M.; Tong L.; Yong S. S.; Li B.; Chaurasia S. S.; Shui G.; Wenk M. R. Meibum Lipid Composition in Asians with Dry Eye Disease. PLoS One 2011, 6 (10), e24339 10.1371/journal.pone.0024339. 22043274 PMC3197196

[ref16] Chen J.; Keirsey J. K.; Green K. B.; Nichols K. K. Expression Profiling of Nonpolar Lipids in Meibum From Patients With Dry Eye: A Pilot Study. Invest. Ophthalmol. Visual Sci. 2017, 58 (4), 2266–2274. 10.1167/iovs.16-20902. 28426869 PMC5398788

[ref17] Miyamoto M.; Sassa T.; Sawai M.; Kihara A. Lipid Polarity Gradient Formed by ω-Hydroxy Lipids in Tear Film Prevents Dry Eye Disease. eLife 2020, 9, e53582 10.7554/eLife.53582. 32252890 PMC7138607

[ref18] Schuett B. S.; Millar T. J. An Investigation of the Likely Role of (O-Acyl) ω-Hydroxy Fatty Acids in Meibomian Lipid Films Using (O-Oleyl) ω-Hydroxy Palmitic Acid as a Model. Exp. Eye Res. 2013, 115, 57–64. 10.1016/j.exer.2013.06.016. 23792170

[ref19] Bland H. C.; Moilanen J. A.; Ekholm F. S.; Paananen R. O. Investigating the Role of Specific Tear Film Lipids Connected to Dry Eye Syndrome: A Study on O -Acyl-ω-Hydroxy Fatty Acids and Diesters. Langmuir 2019, 35 (9), 3545–3552. 10.1021/acs.langmuir.8b04182. 30712353

[ref20] Hancock S. E.; Ailuri R.; Marshall D. L.; Brown S. H. J.; Saville J. T.; Narreddula V. R.; Boase N. R.; Poad B. L. J.; Trevitt A. J.; Willcox M. D. P.; Kelso M. J.; Mitchell T. W.; Blanksby S. J. Mass Spectrometry-Directed Structure Elucidation and Total Synthesis of Ultra-Long Chain (O -Acyl)-ω-Hydroxy Fatty Acids. J. Lipid Res. 2018, 59 (8), 1510–1518. 10.1194/jlr.M086702. 29907595 PMC6071768

[ref21] Butovich I. A.; Wojtowicz J. C.; Molai M. Human Tear Film and Meibum. Very Long Chain Wax Esters and (O-Acyl)-Omega-Hydroxy Fatty Acids of Meibum. J. Lipid Res. 2009, 50 (12), 2471–2485. 10.1194/jlr.M900252-JLR200. 19535818 PMC2781319

[ref22] Balas L.; Bertrand-Michel J.; Viars F.; Faugere J.; Lefort C.; Caspar-Bauguil S.; Langin D.; Durand T. Regiocontrolled Syntheses of FAHFAs and LC-MS/MS Differentiation of Regioisomers. Org. Biomol. Chem. 2016, 14 (38), 9012–9020. 10.1039/C6OB01597B. 27603797

[ref23] Greaves J.; Munro K. R.; Davidson S. C.; Riviere M.; Wojno J.; Smith T. K.; Tomkinson N. C. O.; Chamberlain L. H. Molecular Basis of Fatty Acid Selectivity in the ZDHHC Family of S-Acyltransferases Revealed by Click Chemistry. Proc. Natl. Acad. Sci. U. S. A. 2017, 114 (8), E1365–E1374. 10.1073/pnas.1612254114. 28167757 PMC5338407

[ref24] Girlanda-Junges C.; Keyling-Bilger F.; Schmitt G.; Luu B. Effect of Cyclohexenonic Long Chain Fatty Alcohols on Neurite Outgrowth. Study on Structure-Activity Relationship. Tetrahedron 1998, 54 (27), 7735–7748. 10.1016/S0040-4020(98)00406-2.

[ref25] Abad J.-L.; Fabriàs G.; Camps F. Synthesis of Deuterated Fatty Acids to Investigate the Biosynthetic Pathway of Disparlure, the Sex Pheromone of the Gypsy Moth, Lymantria Dispar. Lipids 2004, 39 (4), 397–401. 10.1007/s11745-004-1244-0. 15357028

[ref26] Gontijo V. S.; Oliveira M. É.; Resende R. J.; Fonseca A. L.; Nunes R. R.; Júnior M. C.; Taranto A. G.; Torres N. M. P. O.; Viana G. H. R.; Silva L. M.; Alves R. B.; Varotti F. P.; Freitas R. P. Long-Chain Alkyltriazoles as Antitumor Agents: Synthesis, Physicochemical Properties, and Biological and Computational Evaluation. Med. Chem. Res. 2015, 24 (1), 430–441. 10.1007/s00044-014-1137-3.

[ref27] Wuts P. G. M.; Greene T. W.; Greene T. W. Greene’s Protective Groups in Organic Synthesis.; John Wiley & Sons, Inc.: Hoboken, N.J., 2007.

[ref28] Maharvi G. M.; Edwards A. O.; Fauq A. H. Chemical Synthesis of Deuterium-Labeled and Unlabeled Very Long Chain Polyunsaturated Fatty Acids. Tetrahedron Lett. 2010, 51 (49), 6426–6428. 10.1016/j.tetlet.2010.09.139. 21076635 PMC2976573

[ref29] Cateni F.; Zacchigna M.; Zilic J.; Di Luca G. Total Synthesis of a Natural Cerebroside from Euphorbiaceae. Helv. Chim. Acta 2007, 90 (2), 282–290. 10.1002/hlca.200790032.

[ref30] He J.; Baldwin J.; Lee V. Studies towards the Synthesis of the Antibiotic Tetrodecamycin. Synlett 2018, 29 (08), 1117–1121. 10.1055/s-0037-1609303.

[ref31] Primdahl K. G.; Tungen J. E.; Aursnes M.; Hansen T. V.; Vik A. An Efficient Total Synthesis of Leukotriene B 4. Org. Biomol. Chem. 2015, 13 (19), 5412–5417. 10.1039/C5OB00473J. 25857248

[ref32] Lee J.-H.; Sirion U.; Jang K.-S.; Lee B.-S.; Chi D. Y. Facile One-Pot Two-Step Hydroxylation of Alkyl Halides and Alkyl Sulfonates via Acetate Intermediates. Bull. Korean Chem. Soc. 2008, 29, 2491–2495. 10.5012/bkcs.2008.29.12.2491.

[ref33] Zemplén G.; Gerecs A.; Hadácsy I. Über Die Verseifung Acetylierter Kohlenhydrate. Ber. Dtsch. Chem. Ges. B 1936, 69 (8), 1827–1829. 10.1002/cber.19360690807.

[ref34] Raghunanan L.; Yue J.; Narine S. S. Synthesis and Characterization of Novel Diol, Diacid and Di-Isocyanate from Oleic Acid. J. Am. Oil Chem. Soc. 2014, 91 (2), 349–356. 10.1007/s11746-013-2376-z.

[ref35] Heilbron I.; Jones E. R. H.; Sondheimer F. 315. Researches on Acetylenic Compounds. Part XIV. A Study of the Reactions of the Readily Available Ethynyl-Ethylenic Alchohol, Pent-2-En-4-Yn-1-Ol. J. Chem. Soc. 1947, 1586–1590. 10.1039/jr9470001586.

[ref36] Laatikainen R.; Tiainen M.; Korhonen S.-P.; Niemitz M.; Harris R. K. Computerized Analysis of High-Resolution Solution-State Spectra. eMagRes 2011, 10.1002/9780470034590.emrstm1226.

[ref37] Ekholm F. S.; Sinkkonen J.; Leino R. Fully Deprotected β-(1→2)-Mannotetraose Forms a Contorted α-Helix in Solution: Convergent Synthesis and Conformational Characterization by NMR and DFT. New J. Chem. 2010, 34 (4), 667–675. 10.1039/b9nj00702d.

[ref38] Ekholm F. S.; Eklund P.; Leino R. A Short Semi-Synthesis and Complete NMR-Spectroscopic Characterization of the Naturally Occurring Lignan Glycoside Matairesinol 4,4′-Di-O-β-d-Diglucoside. Carbohydr. Res. 2010, 345 (13), 1963–1967. 10.1016/j.carres.2010.06.008. 20655039

[ref39] Ekholm F. S.; Schneider G.; Wölfling J.; Leino R. An Approach to the Synthesis and Attachment of Scillabiose to Steroids. Steroids 2011, 76 (6), 588–595. 10.1016/j.steroids.2011.02.010. 21352842

[ref40] Ekholm F. S.; Ardá A.; Eklund P.; André S.; Gabius H.-J.; Jiménez-Barbero J.; Leino R. Studies Related to Norway Spruce Galactoglucomannans: Chemical Synthesis, Conformation Analysis, NMR Spectroscopic Characterization, and Molecular Recognition of Model Compounds. Chem. - Eur. J. 2012, 18 (45), 14392–14405. 10.1002/chem.201200510. 23008171

[ref41] Frost D. J.; Gunstone F. D. The PMR Analysis of Non-Conjugated Alkenoic and Alkynoic Acids and Esters. Chem. Phys. Lipids 1975, 15 (1), 53–85. 10.1016/0009-3084(75)90032-8. 1182930

[ref42] Borchman D.; Foulks G. N.; Yappert M. C.; Milliner S. E. Changes in Human Meibum Lipid Composition with Age Using Nuclear Magnetic Resonance Spectroscopy. Invest. Ophthalmol. Visual Sci. 2012, 53 (1), 475–482. 10.1167/iovs.11-8341. 22169100 PMC3292379

[ref43] Borchman D.; Yappert M. C.; Milliner S. E.; Duran D.; Cox G. W.; Smith R. J.; Bhola R. 13C and 1H NMR Ester Region Resonance Assignments and the Composition of Human Infant and Child Meibum. Exp. Eye Res. 2013, 112, 151–159. 10.1016/j.exer.2013.04.017. 23644094

[ref44] Sledge S.; Henry C.; Borchman D.; Yappert M.; Bhola R.; Ramasubramanian A.; Blackburn R.; Austin J.; Massey K.; Sayied S.; Williams A.; Georgiev G.; Schikler K. Human Meibum Age, Lipid–Lipid Interactions and Lipid Saturation in Meibum from Infants. Int. J. Mol. Sci. 2017, 18 (9), 1862 10.3390/ijms18091862. PMC5618511 28846660

[ref45] Li J.; Vosegaard T.; Guo Z. Applications of Nuclear Magnetic Resonance in Lipid Analyses: An Emerging Powerful Tool for Lipidomics Studies. Prog. Lipid Res. 2017, 68, 37–56. 10.1016/j.plipres.2017.09.003. 28911967

[ref46] Langmuir I.; Schaefer V. J. Rates of Evaporation of Water through Compressed Monolayers on Water. J. Franklin Inst. 1943, 235 (2), 119–162. 10.1016/S0016-0032(43)90904-4.

[ref47] Kaganer V. M.; Möhwald H.; Dutta P. Structure and Phase Transitions in Langmuir Monolayers. Rev. Mod. Phys. 1999, 71 (3), 779–819. 10.1103/RevModPhys.71.779.

[ref48] Nagyová B.; Tiffany J. M. Components Responsible for the Surface Tension of Human Tears. Curr. Eye Res. 1999, 19 (1), 4–11. 10.1076/ceyr.19.1.4.5341. 10415451

[ref49] Chen J.; Nichols K. K. Composition of Diesters in Human Meibum. ARVO Annual Meeting Abstract 2016, Seattle, Wash. Invest. Ophthalmol. Vis. Sci. 2016, 57, ( (12), ), 4818.

[ref50] Nicolaides N.; Kaitaranta J. K.; Rawdah T. N.; Macy J. I.; Boswell F. M. 3rd; Smith R. E. Meibomian Gland Studies: Comparison of Steer and Human Lipids. Invest. Ophthalmol. Vis. Sci. 1981, 20 (4), 522–536. 7194326

[ref51] Paananen R. O.; Viitaja T.; Olżyńska A.; Ekholm F. S.; Moilanen J.; Cwiklik L. Interactions of Polar Lipids with Cholesteryl Ester Multilayers Elucidate Tear Film Lipid Layer Structure. Ocul Surf. 2020, 18 (4), 545–553. 10.1016/j.jtos.2020.06.001. 32562857

[ref52] Deo A. V.; Kulkarni S. B.; Gharpurey M. K.; Biswas A. B. On the Rate of Spreading of Long Chain N-Alcohols and n-Alkoxy Ethanols from Solid into Monolayer on Water. J. Colloid Sci. 1964, 19 (9), 820–830. 10.1016/0095-8522(64)90058-3.

[ref53] La Mer V. K.; Healy T. W.; Aylmore L. A. G. The Transport of Water through Monolayers of Long-Chain n-Paraffinic Alcohols. J. Colloid Sci. 1964, 19 (8), 673–684. 10.1016/0095-8522(64)90075-3.

[ref54] King-Smith P. E.; Bailey M. D.; Braun R. J. Four Characteristics and a Model of an Effective Tear Film Lipid Layer (TFLL). Ocul Surf. 2013, 11 (4), 236–245. 10.1016/j.jtos.2013.05.003. 24112227 PMC4313865

[ref55] Borchman D.; Ramasubramanian A.; Foulks G. N. Human Meibum Cholesteryl and Wax Ester Variability With Age, Sex, and Meibomian Gland Dysfunction. Invest. Ophthalmol. Vis. Sci. 2019, 60 (6), 2286–2293. 10.1167/iovs.19-26812. 31112994 PMC6530518

[ref56] Laatikainen R.; Niemitz M.; Weber U.; Sundelin J.; Hassinen T.; Vepsäläinen J. General Strategies for Total-Lineshape-Type Spectral Analysis of NMR Spectra Using Integral-Transform Iterator. J. Magn. Reson., Ser. A 1996, 120 (1), 1–10. 10.1006/jmra.1996.0094.

