# Correction to The Properties and Role of *O*-Acyl-ω-hydroxy Fatty Acids and Type I-St and Type II Diesters in the Tear Film Lipid Layer Revealed by a Combined Chemistry and Biophysics Approach

**DOI:** 10.1021/acs.joc.1c02513

**Published:** 2021-11-10

**Authors:** Tuomo Viitaja, Jan-Erik Raitanen, Jukka Moilanen, Riku O. Paananen, Filip S. Ekholm

Since the publication of the original article, we have become aware of additional previous studies on the synthesis of model type I-St (Butovich et al. Sci. Rep.2011, 1, 2422355543) and type II diesters (Butovich et al. Invest. Ophthalmol. Vis. Sci.2012, 53, 6881−68962291862910.1167/iovs.12-10516PMC3466071), which were not cited in the original paper. All conclusions in our submission are unchanged, including original and expanded characterization data.

