# Supplementary material for: The Properties and Role of O-Acyl-ω-hydroxy Fatty Acids and Type I-St and Type II Diesters in the Tear Film Lipid Layer Revealed by a Combined Chemistry and Biophysics Approach
Source: J Org Chem. 2021 Mar 17;86(7):4965–76. doi: 10.1021/acs.joc.0c02882 (PMC8041317; doi:10.1021/acs.joc.0c02882)
Supplement: Supplementary file 1 — jo0c02882_si_001.pdf [file jo0c02882_si_001.pdf]

Supporting Information

The Properties and Role of *O*-Acyl-ω-hydroxy Fatty Acids and Type I-St and Type II Diesters in the Tear Film Lipid Layer Revealed by a Combined Chemistry and Biophysics Approach

Tuomo Viitaja,[1,2†] Jan-Erik Raitanen,[1†] Jukka Moilanen,[2] Riku O. Paananen,[1,2*] Filip S. Ekholm[1*]

[1]Department of Chemistry, University of Helsinki, P.O. Box 55, FI-00014 Helsinki, Finland

[2]Ophthalmology, University of Helsinki and Helsinki University Hospital, Haartmaninkatu 8, FI-00290 Helsinki, Finland

[†]Equal contribution

*** Corresponding author contact:** riku.o.paananen@helsinki.fi, filip.ekholm@helsinki.fi

Table of contents

1. Numbering of Molecules……………………………………………………………………………S2

2. NMR Spectra of Synthesized Compounds…………………………………………………………S2

3. Supporting Material Related to the Biophysical Experiments…………………………………..S13

1. Numbering of Molecules

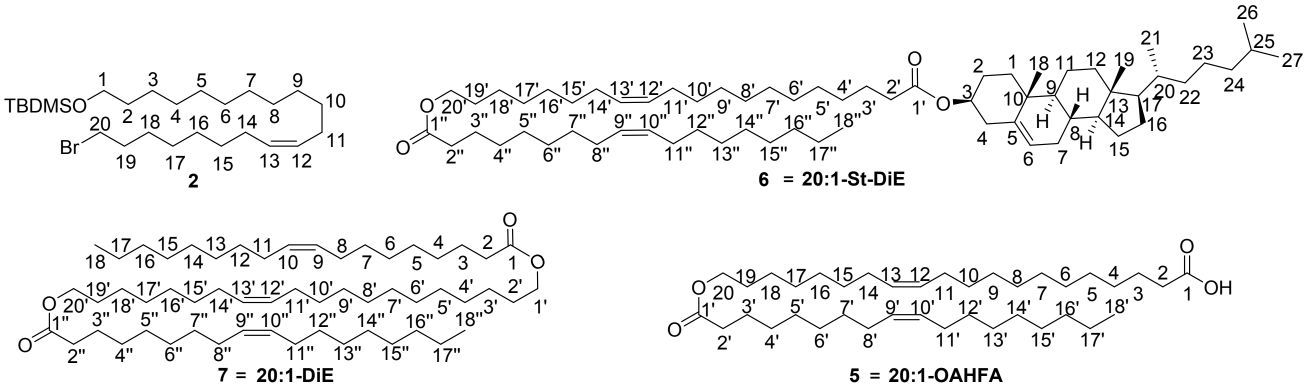

Figure S1. Selected examples showcasing the numbering employed in the NMR spectroscopic characterization data reported in the experimental section.

2. NMR Spectra of Synthesized Compounds

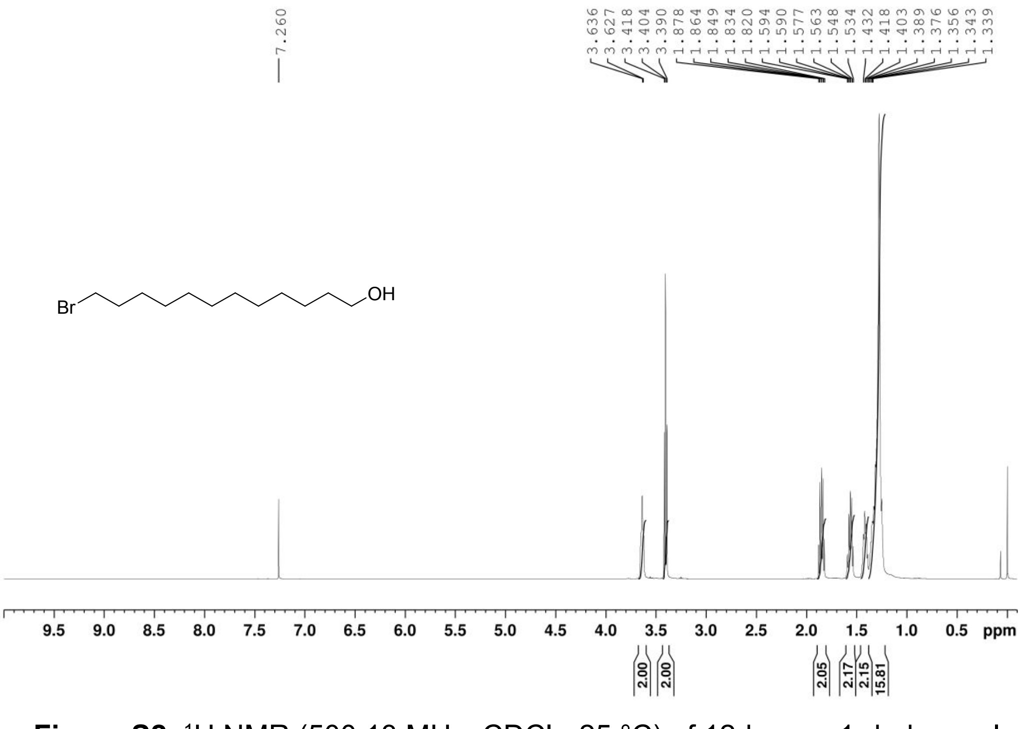

Figure S2. ^{1}H NMR (500.13 MHz, $CDCl_3$, 25 °C) of 12-bromo-1-dodecanol.

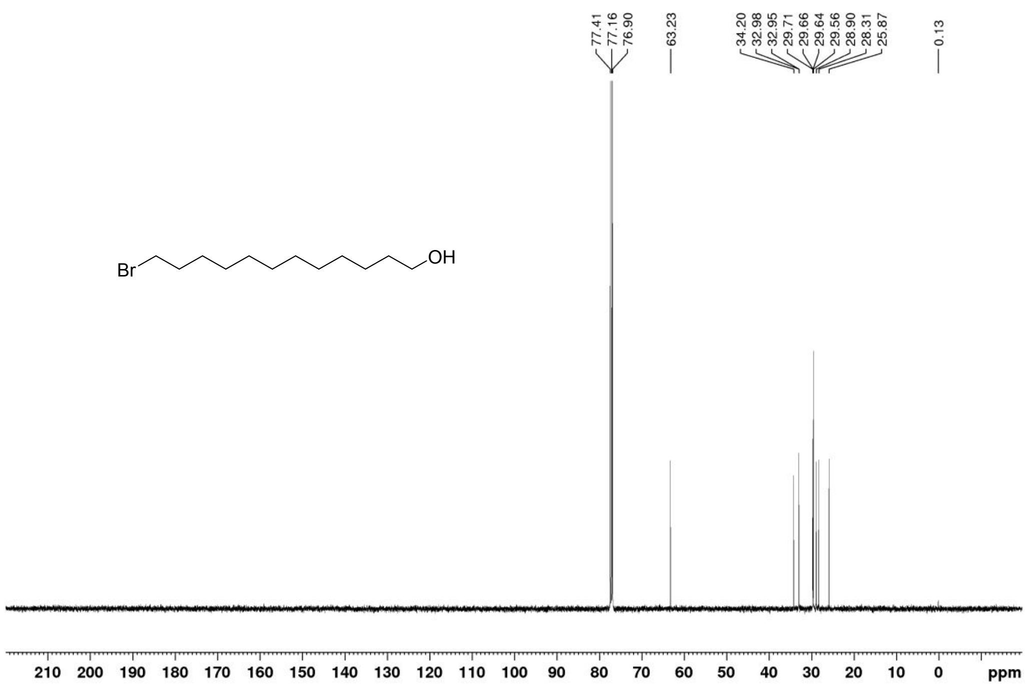

Figure S3. $^{13}C\{^1H\}$ NMR (125.68 MHz, $CDCl_3$, 25 °C) of 12-bromo-1-dodecanol.

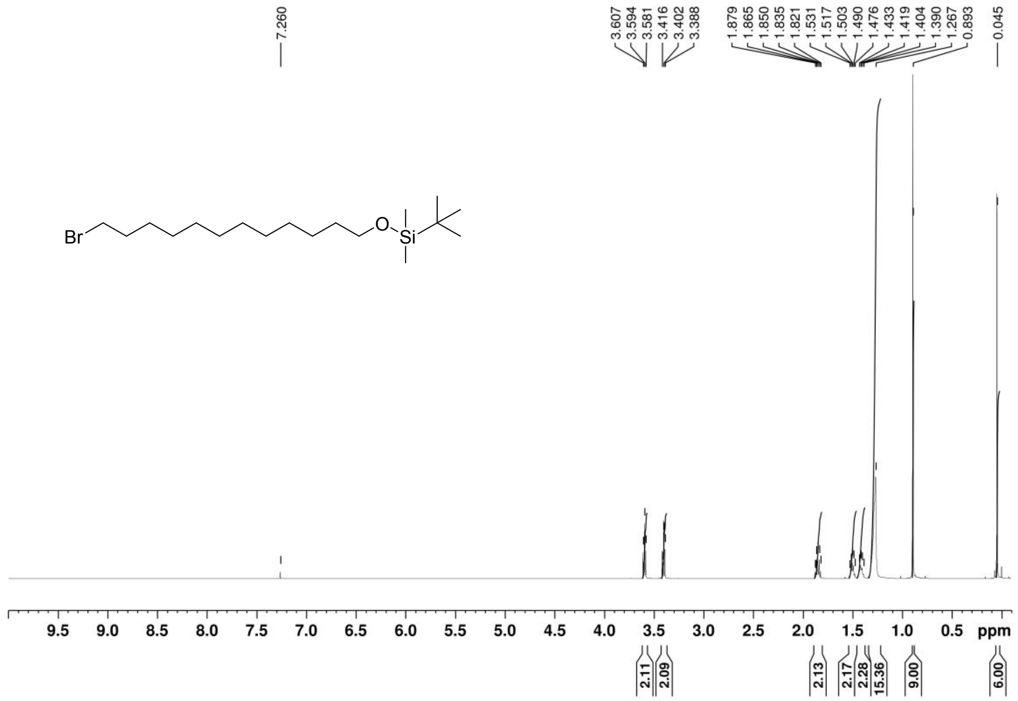

Figure S4. ^1H NMR (500.13 MHz, $CDCl_3$, 25 °C) of **1**.

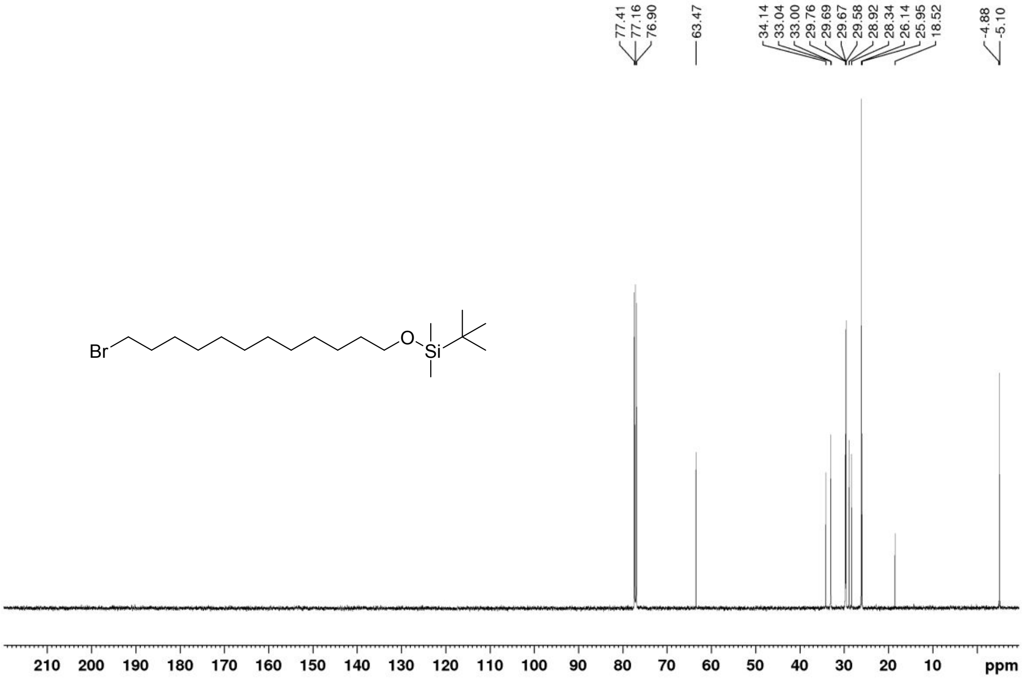

Figure S5. $^{13}C\{^{1}H\}$ NMR (125.68 MHz, $CDCl_3$, 25 °C) of **1**.

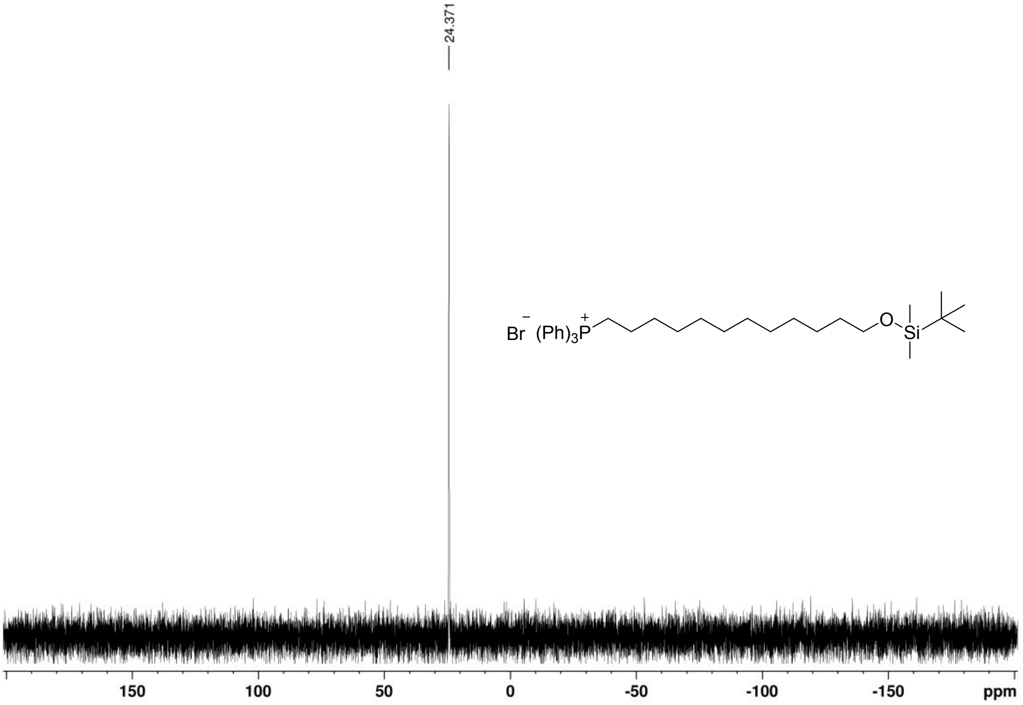

Figure S6. ^{31}P NMR (202.4 MHz, $CDCl_3$, 25 °C) of Fragment B.

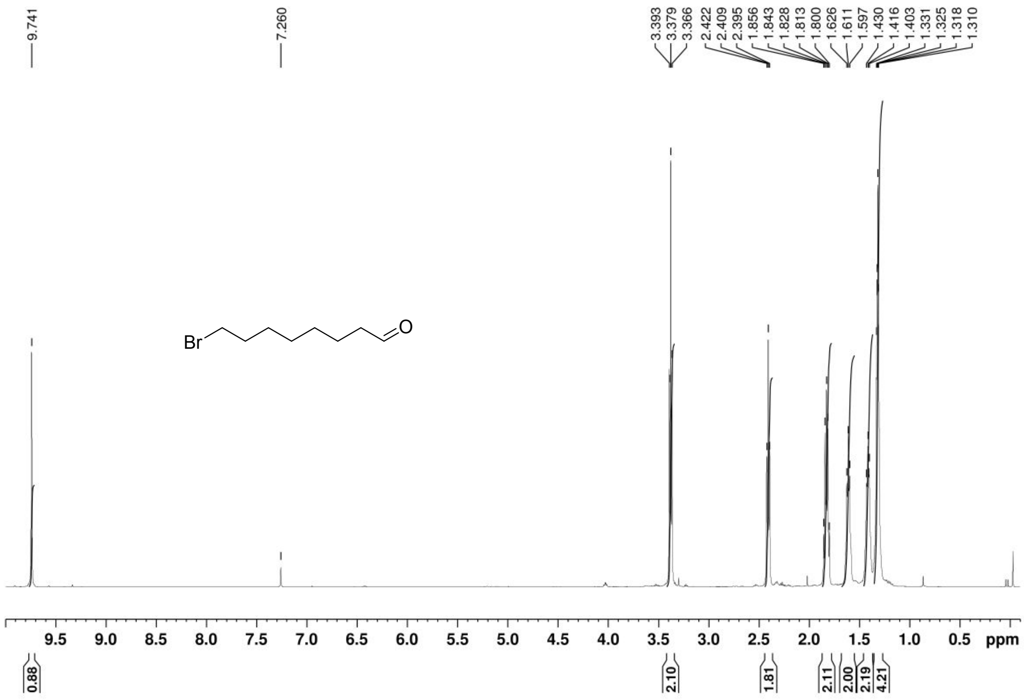

Figure S7. ^1H NMR (500.13 MHz, $CDCl_3$, 25 °C) of Fragment A.

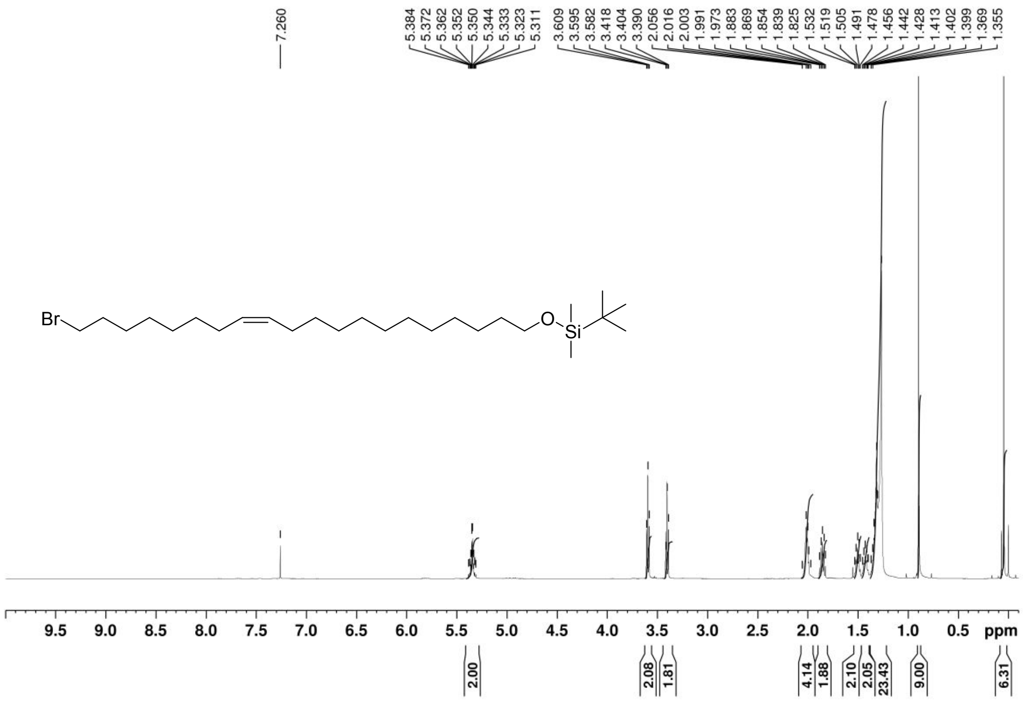

Figure S8. ^1H NMR (499.82 MHz, $CDCl_3$, 25 °C) of **2**.

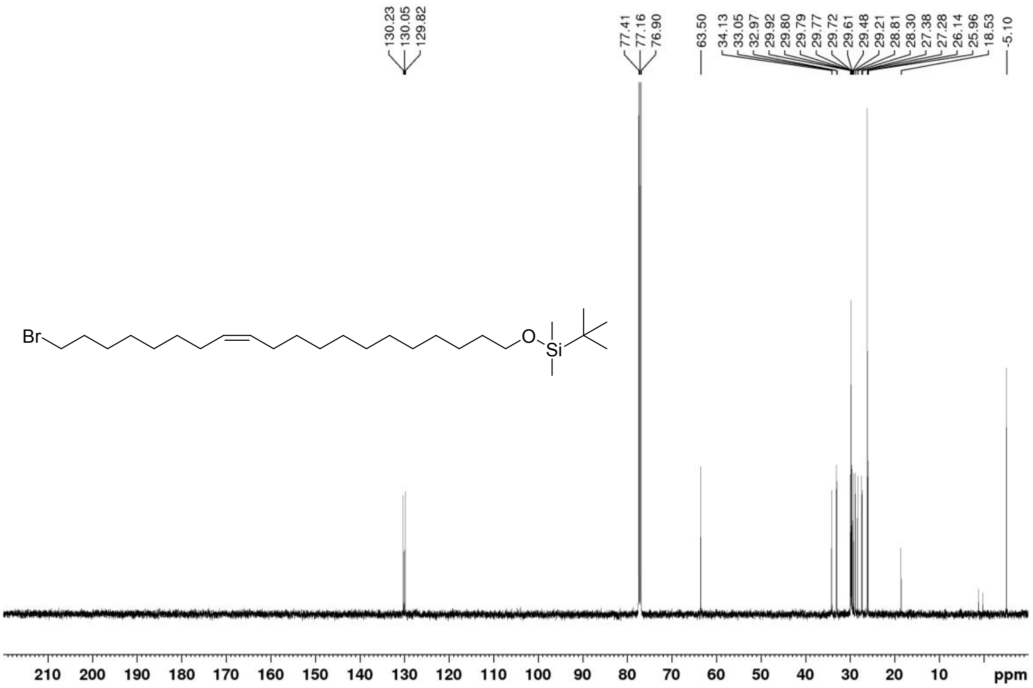

Figure S9. $^{13}C\{^1H\}$ NMR (125.68 MHz, $CDCl_3$, 25 °C) of **2**.

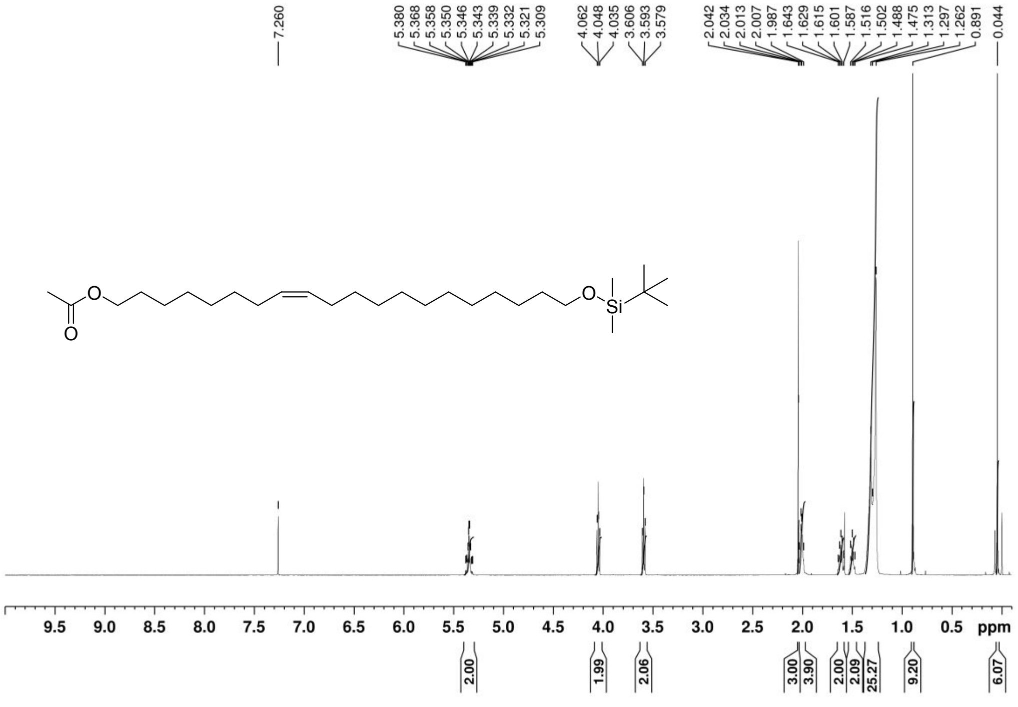

Figure S10. ^1H NMR (500.13 MHz, $CDCl_3$, 25 °C) of (12*Z*)-20-acetoxy-1-*tert*-butyldimethylsilyloxyeicos-12-ene.

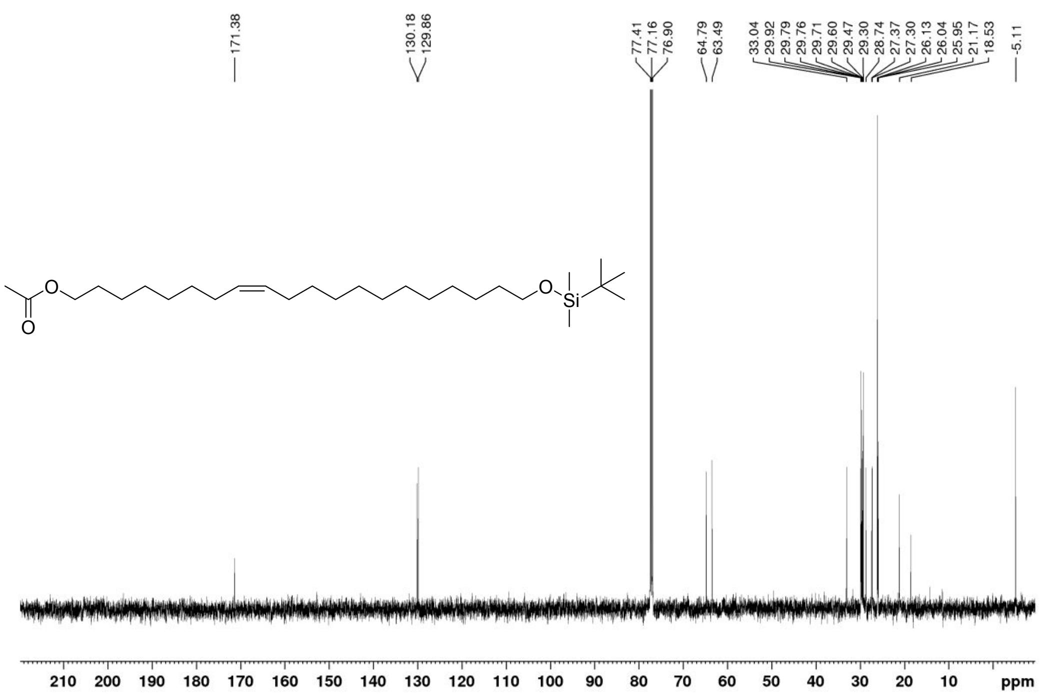

Figure S11. $^{13}C\{^{1}H\}$ NMR (125.68 MHz, $CDCl_3$, 25 °C) of (12*Z*)-20-acetoxy-1-*tert*-butyldimethylsilyloxyeicos-12-ene.

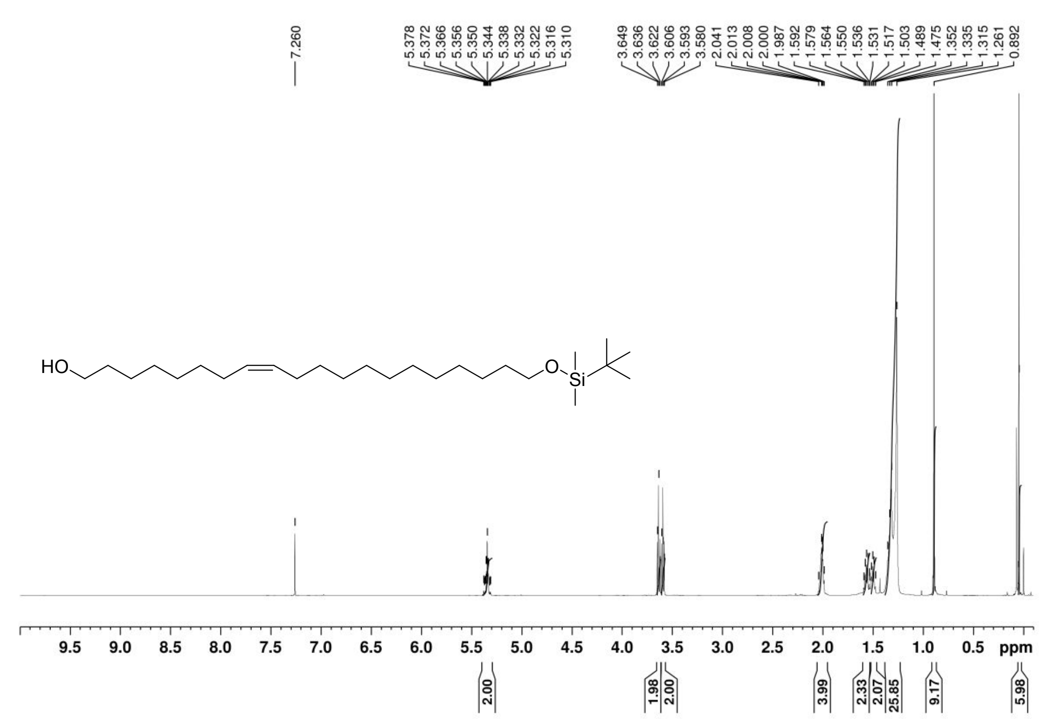

Figure S12. ^{1}H NMR (500.13 MHz, $CDCl_3$, 25 °C) of **3**.

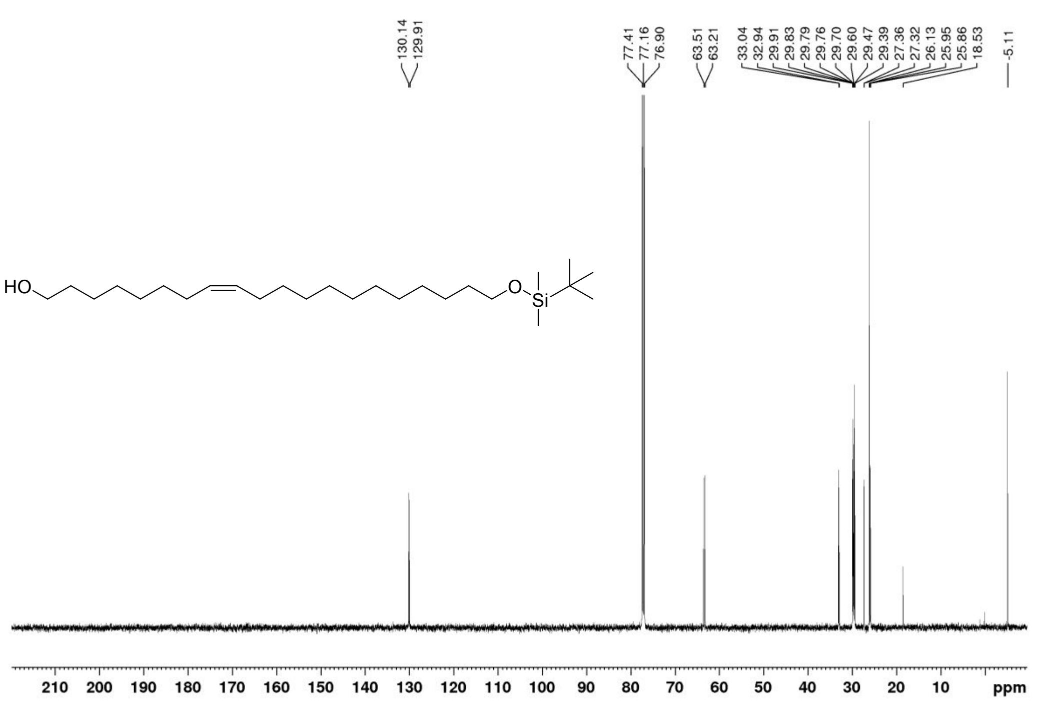

Figure S13. $^{13}C\{^1H\}$ NMR (125.68 MHz, $CDCl_3$, 25 °C) of **3**.

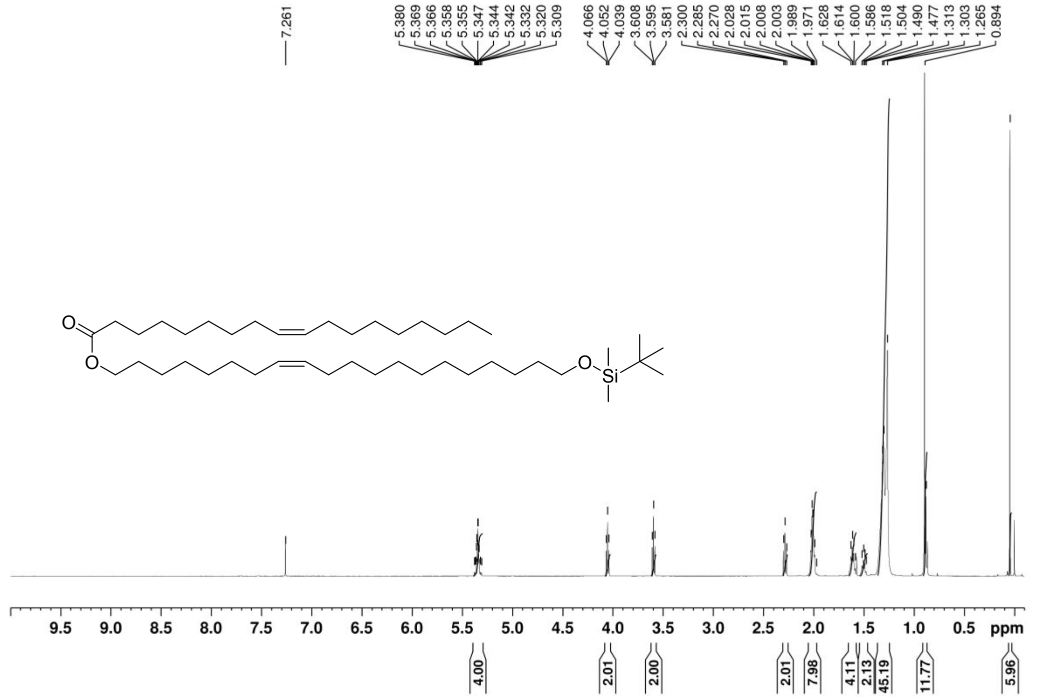

Figure S14. ^1H NMR (500.13 MHz, $CDCl_3$, 25 °C) of (12*Z*)-20-oleoyloxy-1-*tert*-butyldimethylsilyloxyeicos-12-ene.

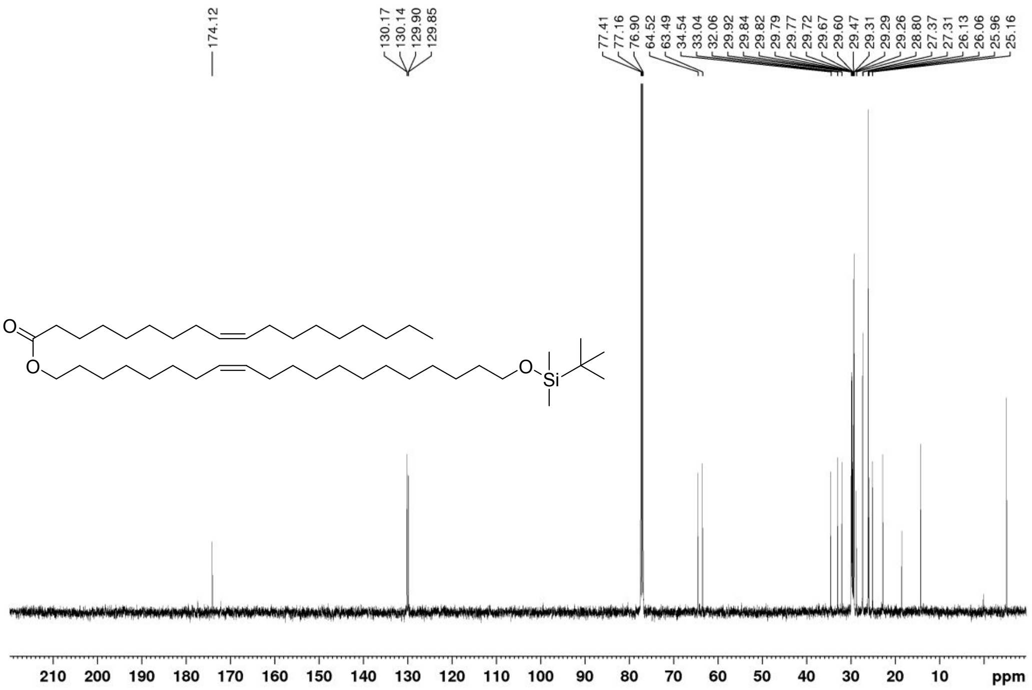

Figure S15. $^{13}C\{^1H\}$ NMR (125.68 MHz, $CDCl_3$, 25 °C) of (12*Z*)-20-oleoyloxy-1-*tert*-butyldimethylsilyloxyeicos-12-ene.

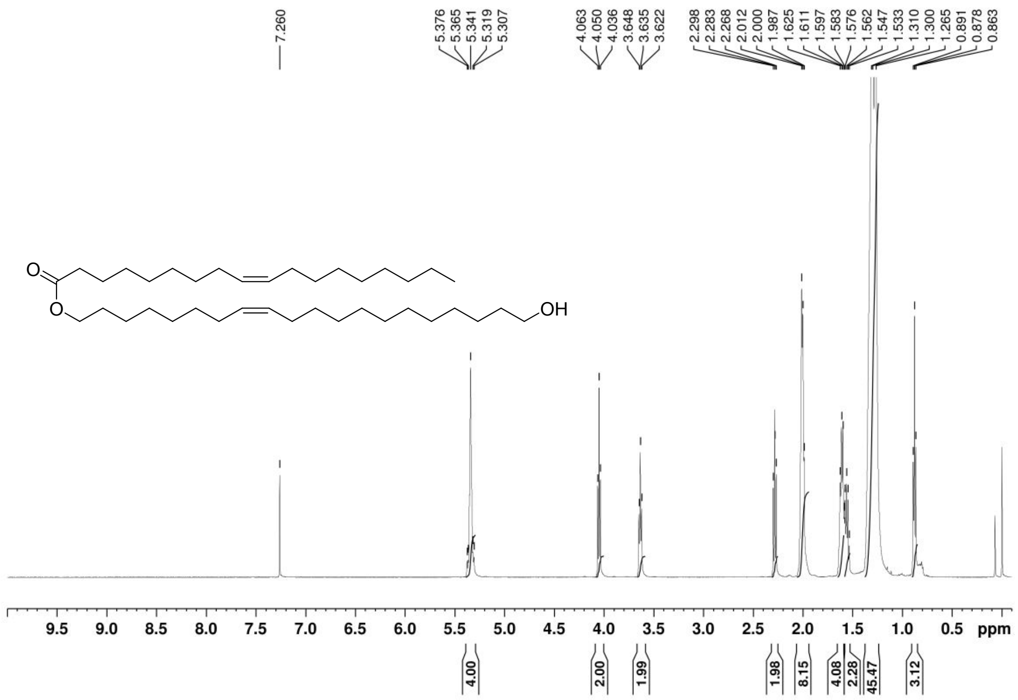

Figure S16. ^1H NMR (500.13 MHz, $CDCl_3$, 25 °C) of **4**.

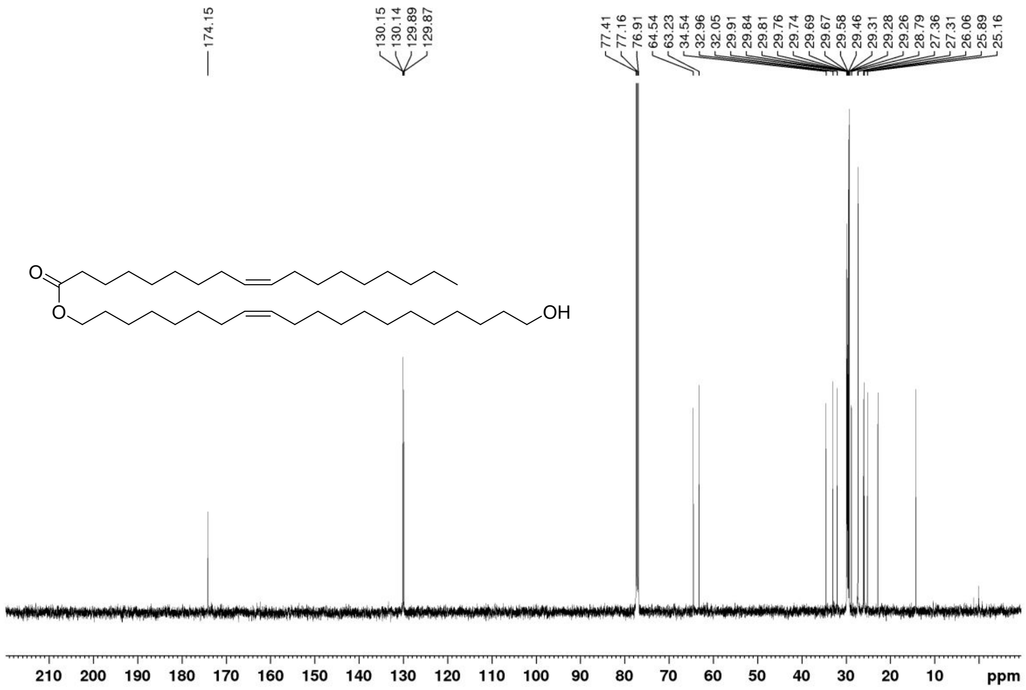

Figure S17. $^{13}C\{^{1}H\}$ NMR (125.68 MHz, $CDCl_3$, 25 °C) of **4**.

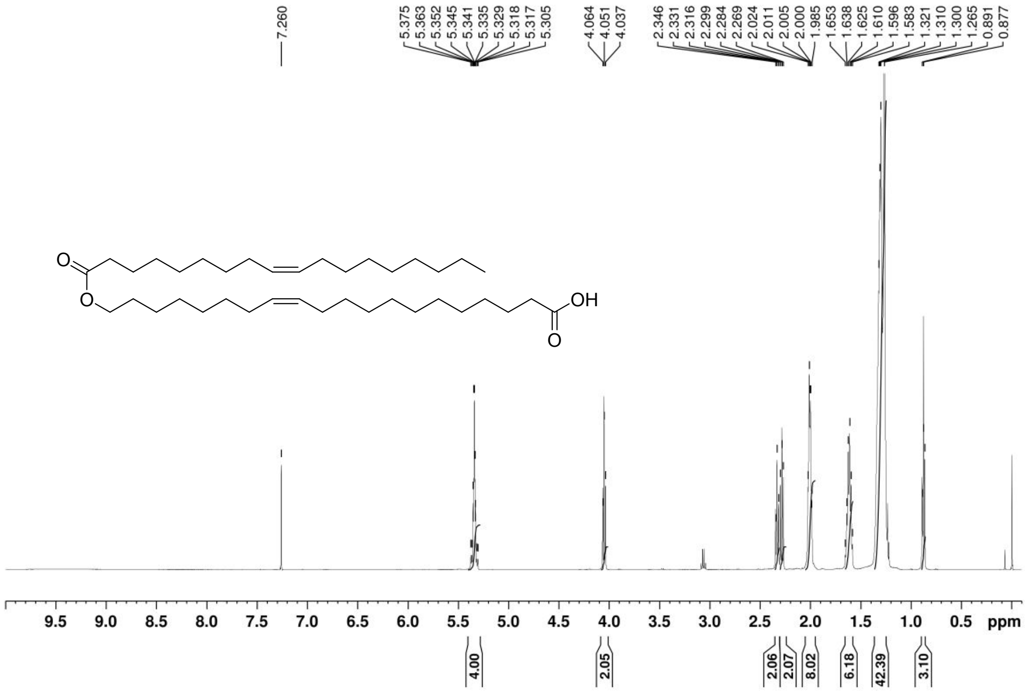

Figure S18. ^{1}H NMR (500.13 MHz, $CDCl_3$, 25 °C) of **5**.

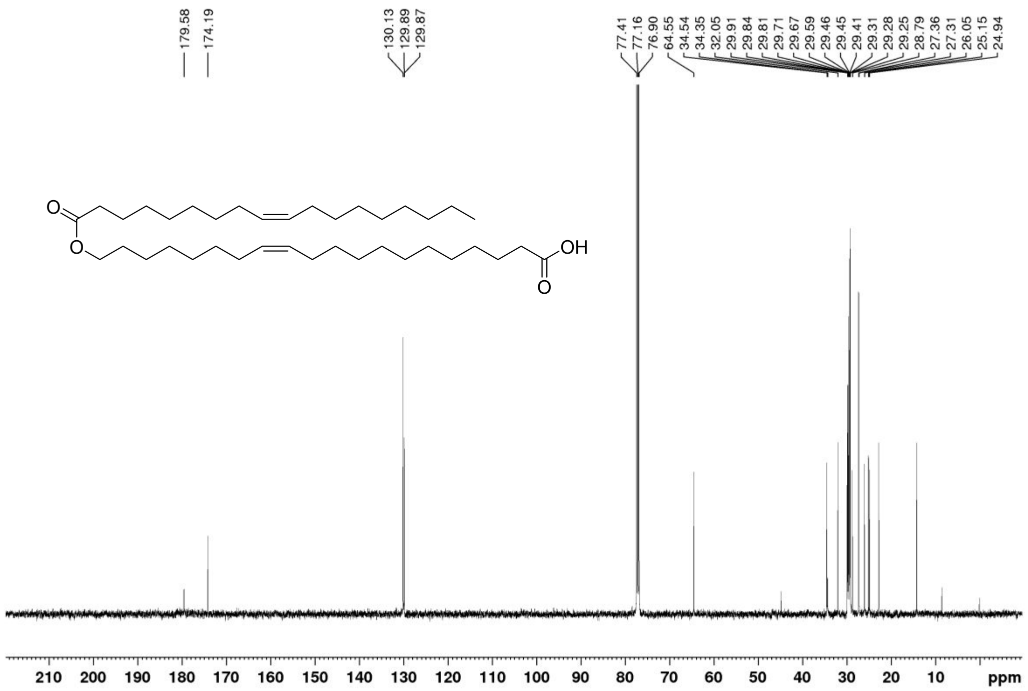

Figure S19. $^{13}C\{^{1}H\}$ NMR (125.68 MHz, $CDCl_3$, 25 °C) **5**.

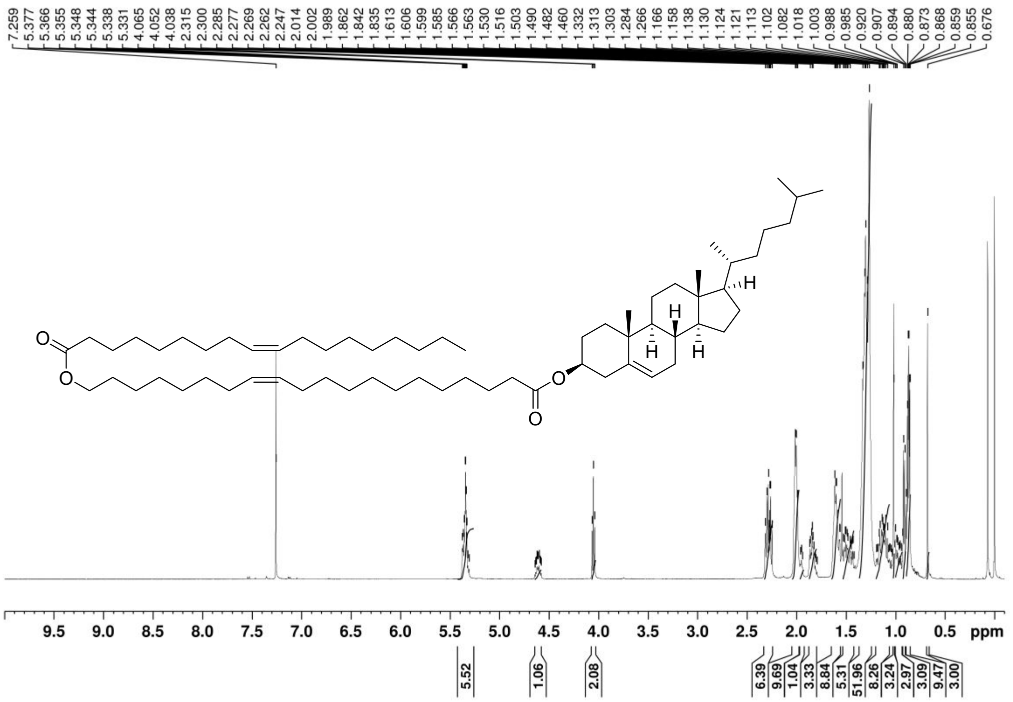

Figure S20. ^{1}H NMR (499.82 MHz, $CDCl_3$) of **6**.

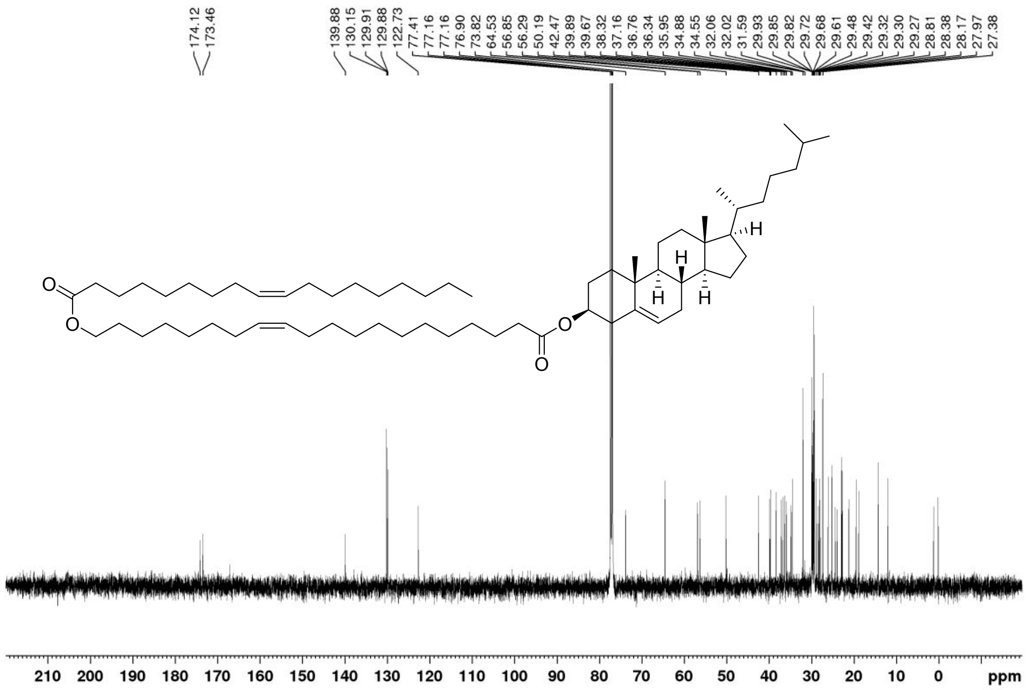

Figure S21. $^{13}C\{^{1}H\}$ NMR (125.68 MHz, $CDCl_3$) of **6**.

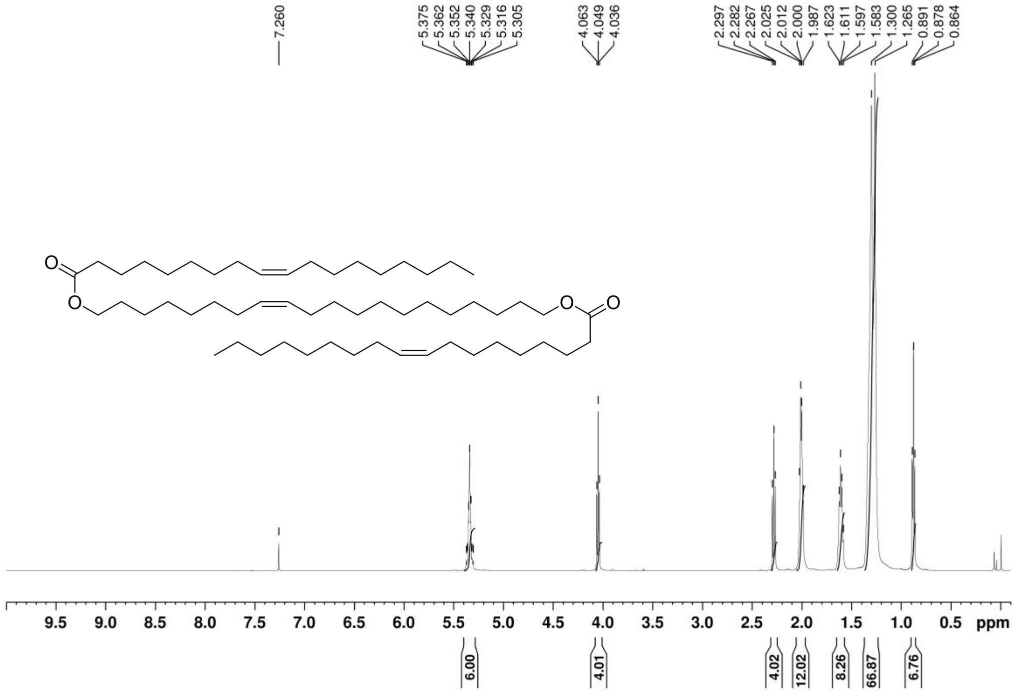

Figure S22. ^{1}H NMR (499.82 MHz, $CDCl_3$) of **7**.

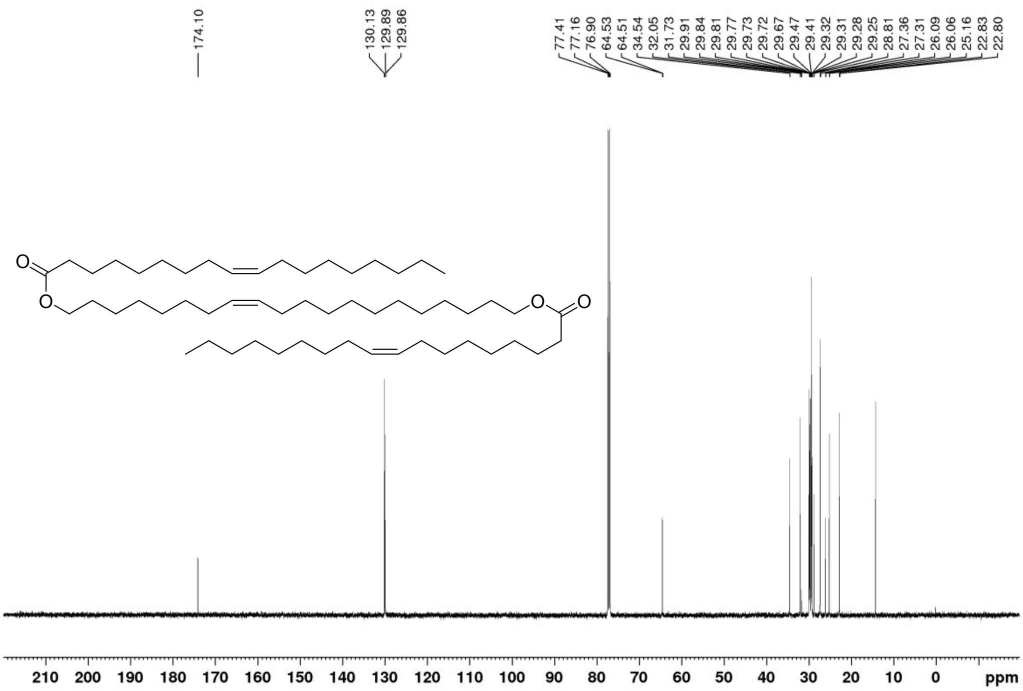

Figure S23. $^{13}C\{^1H\}$ NMR (125.68 MHz, $CDCl_3$, 25 °C) of **7**.

3. Supporting Material Related to the Biophysical Experiments

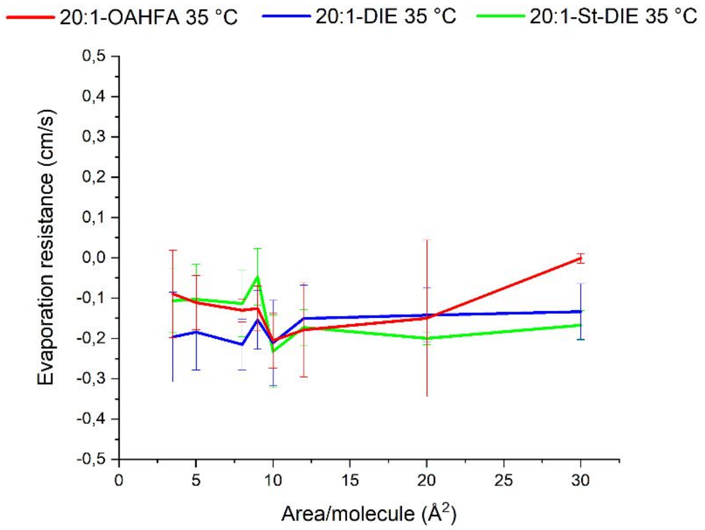

Figure S24. Evaporation resistance of synthesized lipids as a function of mean molecular area.

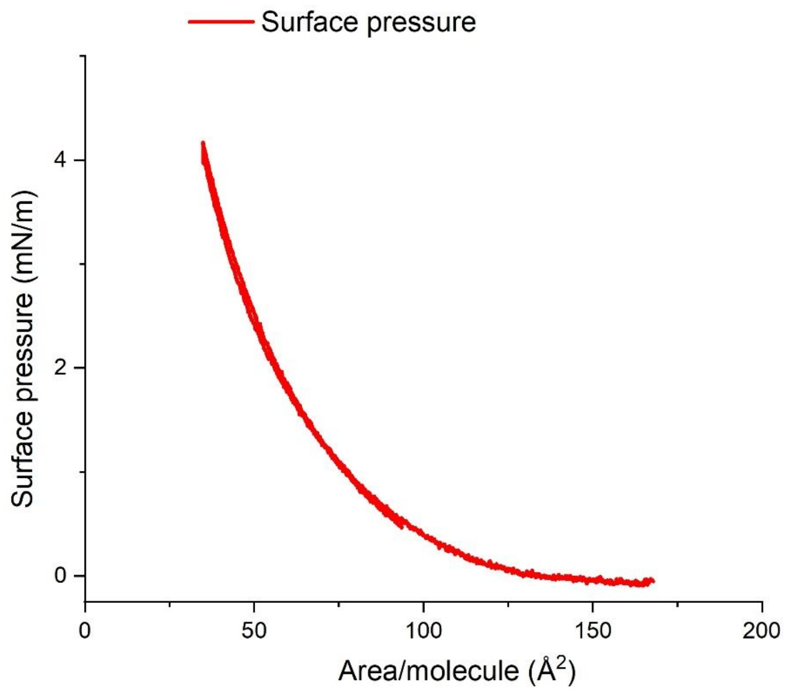

Figure S25. 20:1-St-DiE surface pressure isotherm at 40 °C.

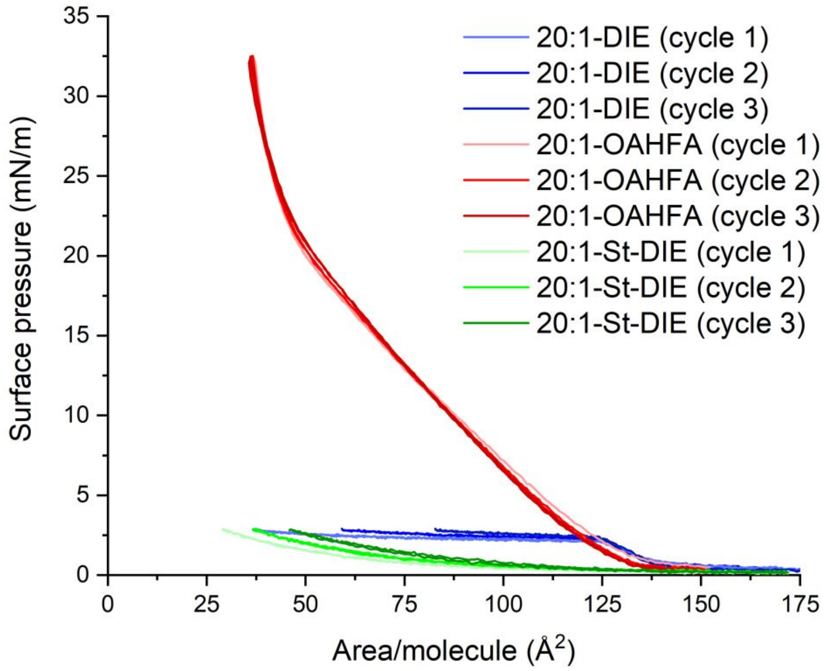

Figure S26. Compression-expansion cycles of studied lipids at 35 °C. The films were compressed three times to surface pressure of either 32 mN/m (20:1-OAHFA) or 3 mN/m (20:1-DIE and 20:1-St-DIE).
